# Western diet triggers cardiac dysfunction in heterozygous *Mybpc3*-targeted knock-in mice: A two-hit model of hypertrophic cardiomyopathy

**DOI:** 10.1016/j.jmccpl.2023.100050

**Published:** 2023-09-19

**Authors:** Edgar E. Nollet, Sila Algül, Max Goebel, Saskia Schlossarek, Nicole N. van der Wel, Judith J.M. Jans, Mark A. van de Wiel, Jaco C. Knol, Thang V. Pham, Sander R. Piersma, Richard de Goeij-de Haas, Jill Hermans, Jan Bert van Klinken, Michel van Weeghel, Riekelt H. Houtkooper, Lucie Carrier, Connie R. Jimenez, Diederik W.D. Kuster, Jolanda van der Velden

**Affiliations:** a Department of Physiology, Amsterdam UMC, Amsterdam, the Netherlands; b Amsterdam Cardiovascular Sciences, Heart failure & Arrhythmias, Amsterdam, the Netherlands; c Institute of Experimental Pharmacology and Toxicology, University Medical Center Hamburg-Eppendorf, Hamburg, Germany; d DZHK (German Centre for Cardiovascular Research), partner site Hamburg/Kiel/Lübeck, University Medical Center Hamburg-Eppendorf, Hamburg, Germany; e Department of Medical Biology, Electron Microscopy Centre, Amsterdam UMC, Amsterdam, the Netherlands; f Department of Genetics, University Medical Center Utrecht, Utrecht University, Utrecht, the Netherlands; g Department of Epidemiology and Data Science, Amsterdam UMC, Amsterdam, the Netherlands; h Department of Medical Oncology, VUmc Cancer Center Amsterdam, OncoProteomics Laboratory, Amsterdam UMC, Amsterdam, the Netherlands; i Laboratory Genetic Metabolic Diseases, Amsterdam UMC, Amsterdam, the Netherlands; j Core Facility Metabolomics, Amsterdam UMC, Amsterdam, the Netherlands; k Amsterdam Gastroenterology Endocrinology and Metabolism institute, Amsterdam, the Netherlands

**Keywords:** Hypertrophic cardiomyopathy, Sarcomere mutation, Metabolic health, Cardiac metabolism, Pathophysiology

## Abstract

**Background and aim:**

Phenotypic expression of hypertrophic cardiomyopathy (HCM) and disease course are associated with unfavorable metabolic health. We investigated if Western diet (WD) feeding is sufficient to trigger cardiac hypertrophy and dysfunction in heterozygous (HET) *Mybpc3*_c.772G>A_ knock-in mice.

**Methods and results:**

Wild-type (WT) and HET mice (3-months-old) were fed a WD or normal chow (NC) for 8 weeks. Metabolomic analyses on serum revealed systemic metabolic derailment in WD-fed WT and HET mice. Strikingly, only WD-fed HET mice developed cardiac hypertrophy and dysfunction, which was not driven by aggravated cardiac myosin binding protein-C haploinsufficiency. WD reduced oxidative phosphorylation and increased toxic lipids in the heart irrespective of genotype. Cardiac proteomic analyses revealed higher abundance of proteins involved in fatty acid oxidation in WD-fed mice, however this increase was blunted in HET compared to WT mice. Accordingly, cardiac metabolomic and lipidomic analyses showed accumulation of acylcarnitines in WD-fed HET vs WT mice.

**Conclusion:**

WD feeding triggered cardiac dysfunction and hypertrophy in otherwise phenotype-negative HET *Mybpc3*_c.772G>A_ mice. We propose that the presence of a HCM mutation predisposes the heart to metabolic inflexibility when subjected to systemic metabolic stress. Our study represents a novel approach to study the interplay between unfavorable metabolic health and mutation-induced defects in HCM disease development.

## Highlights

• Hypertrophic cardiomyopathy is adversely modified by unfavorable metabolic health.
• We mimicked this by feeding a Western diet to asymptomatic heterozygous mice.
• Only Western diet-fed heterozygous mice showed cardiac hypertrophy and dysfunction.
• This was characterized by a blunted upregulation of fatty acid oxidation proteins.
• Our work represents a human-like multifactorial hypertrophic cardiomyopathy model.

## Graphical abstract

**Figure:**
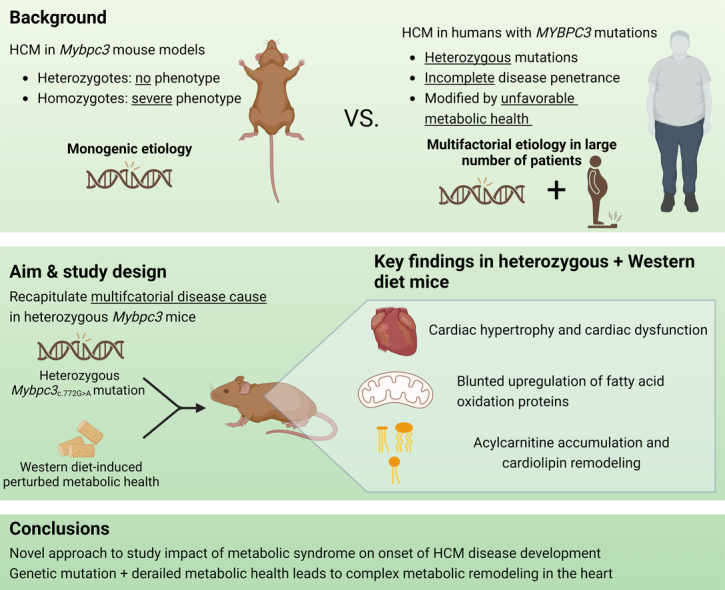

## 1 Introduction

Hypertrophic cardiomyopathy (HCM) is the most common inherited cardiomyopathy, affecting up to 1:200 individuals [1]. Disease hallmarks of HCM include left ventricular (LV) outflow tract obstruction due to septal hypertrophy and diastolic dysfunction [2]. In 50 % of all patients with HCM a heterozygous (HET) pathogenic variant, i.e. mutation, is found in genes encoding sarcomere proteins, the most frequently affected gene being *MYBPC3,* which encodes the thick-filament protein cardiac myosin binding protein-C (cMyBP-C) [[3], [4], [5]]. Truncating mutations represent the vast majority of *MYBPC3* mutations, typically resulting in cMyBP-C haploinsufficiency, which is thought to be the primary pathomechanism induced by such mutations [6]. The causative role of sarcomere mutations in HCM pathogenesis is well-accepted [7], as mutant sarcomere proteins alter cardiomyocyte function and mice with a homozygous *Mybpc3* mutation display severe cardiac remodeling and dysfunction at young age [[8], [9], [10], [11], [12], [13]]. However, there is substantial variation in disease penetrance and clinical course among HET carriers of known disease-causing variants [14], ranging from individuals remaining asymptomatic throughout life to onset of severe disease at young age [15]. HCM mouse models with HET truncating *Mybpc3* mutations do not develop overt disease under normal conditions despite altered myofilament function and subtle structural changes of the heart [[9], [10], [11], [12], [13],[16], [17], [18]], and thus do not recapitulate the human HCM clinical phenotype. The finding that the presence of a HET mutation is solely not sufficient to trigger disease indicates that HCM development is subject to modification by disease-modifying factors. Reports from recent years suggested that unfavorable metabolic health (e.g. elevated body weight, dyslipidemia, hyperglycemia, (pre)diabetes, systemic inflammation) is associated with increased disease penetrance and worsens disease course in patients with HCM [[19], [20], [21], [22], [23], [24], [25], [26]]. This could be mediated by aggravation of mutation-mediated myofilament dysfunction and/or by myocardial changes independent of the pathogenic gene variant [27].

To study if and how perturbed metabolic health triggers development of HCM, phenotype-negative *Mybpc3*-targeted knock-in mice harboring a HET mutation on the last nucleotide of exon 6 (c.772G>A) were subjected to Western diet (WD) feeding for 8 weeks [16]. We then performed a multi-omics approach combined with analyses of mitochondrial function and morphology to extensively characterize metabolic alterations. We report that WD-feeding reduced cardiac mitochondrial respiration and increased cardiac toxic lipid levels. Only in HET mice WD-feeding caused cardiac hypertrophy and dysfunction. HET WD hearts were characterized by an altered cardiolipin pool and blunted upregulation of proteins involved in cardiac fatty acid oxidation compared to hearts from WD-fed wild-type (WT) mice. Here, we provide evidence that a heterozygous sarcomere mutation predisposes the heart to develop hypertrophy and dysfunction in response to perturbed systemic metabolic health. Our work represents a novel approach to study the complex interplay between systemic metabolic stress and mutation-induced defects in HCM disease development.

## 2 Methods

An extended methods section is provided in the Supplementary files.

### 2.1 Animal experiments: Western diet and echocardiography

**Fig. 1:**
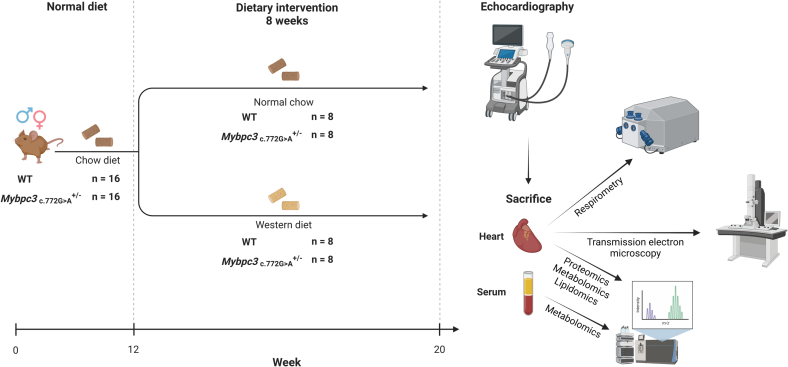
Overview of the timeline and methods applied in this study. Wildtype (WT) and heterozygous *Mybpc3*_*c*.772G>A_ mice were fed normal chow or a Western diet for 8 weeks (n = 8 mice in each group). Echocardiography, respirometry, transmission electron microscopy and serum metabolomics were performed on (left ventricular cardiac tissue of) all mice. Proteomics, metabolomics and lipidomics were performed on left ventricular cardiac tissue samples of 7 WT normal chow-fed mice and 8 mice per group in all other experimental conditions.

Animal experiments were performed in accordance with the Guide for the Animal Care and Use Committee of the VU University Medical Center and with approval of the Animal Care Committee of the VU University Medical Center (CCD-number AVD114002016700) and conform the guidelines from Directive 2010/63/EU of the European Parliament on the protection of animals used for scientific purposes. In total, 32 12-week-old mice were used. Previously generated WT and HET *Mybpc3*-targeted knock-in Black Swiss mice were fed a WD (RD Western Diet D12079B, Research Diets) or normal chow (NC; Teklad Global 2016, Envigo) ad libitum for 8 weeks (*N* = 8 per group, 4 males and 4 females) [11]. Normal chow contained 3000 kcal/kg, 22 % of which derived from protein, 12 % from fat (50 % linoleic, 17.5 % oleic, 12.5 % palmitic, 2.5 % stearic, 2.5 % linolenic and 15 % other fatty acids) and 66 % from carbohydrates (wheat and corn starch). WD contained 4700 kcal/kg, 17 % of which derived from protein, 41 % from fat (27 % palmitic, 27 % oleic, 12.5 % stearic, 10 % myristic, 5.5 % linoleic and 18 % other fatty acids) and 43 % derived from carbohydrates (70 % sucrose, 20 % maltodextrin and 10 % corn starch). Echocardiography (Vevo 2100, Visualsonics) to determine cardiac function was performed on non-fasted mice as described [17] using 2 % isolflurane. Isoflurane dose was raised to 4 % and animals were then euthanized by excising the heart. An overview of the study timeline and methods used is provided in Fig. 1.

### 2.2 Serum metabolomics

Blood was collected from the thorax after excision of the heart, and allowed to clot for 1 h at 37 °C and left to contract overnight at 4 °C. The serum was obtained through centrifugation for 20 min at 2700*g* at 4 °C and stored at −80 °C until further use. Analysis of metabolites was performed as previously described [28]. In brief, a direct-infusion high-resolution mass spectrometry-based metabolomics method was applied in combination with a nano-electrospray ionization source. The mean peak intensities of technical triplicates were calculated. Annotation was done by matching the mass-to-charge ratio of these mass peaks with a range of two parts per million to metabolite masses from the Human Metabolome Database [29], hence putatively identifying metabolites.

### 2.3 Mitochondrial function

Mitochondrial function was assessed using respirometry as described previously [30]. A detailed description is provided in the Supplementary files.

### 2.4 Transmission electron microscopy

Small pieces (2–4 mm^3^) of LV tissue were fixed overnight at room temperature in 2.5 % (vol/vol) glutaraldehyde, 2 % (vol/vol) paraformaldehyde in 0.1 M phosphate buffer and preserved at 4 °C in 1 % (vol/vol) paraformaldehyde until further use. Samples were washed in 0.1 M sodium cacodylate and H_2_O (10 min each) and stained in 1 % (wt/vol) osmium tetroxide for 1 h. Samples were washed overnight in H_2_O and dehydrated stepwise using increasing concentrations of ethanol (3 × 15 min 70 %; 3 × 20 min 80 %; 3 × 20 min 90 %; 3 × 20 min 96 %; 3 × 30 min 100 %). Samples were then placed in propylene oxide for 45 min, incubated overnight in 1:1 propylene oxide/Epon812 and subsequently for 3 h in 1:2 propylene oxide/Epon 812. Samples were then transferred to pure Epon812 for 3 h and embedded in molds with fresh Epon812 and set to polymerize for 3 days at 60 °C. Ultrathin sections (60 nm) were cut using a Leica Ultracut EM UC7 and collected on 100-mesh Formavar-coated copper grids and contrasted by staining for 5 min with 3.5 % (wt/vol) uranyl acetate and 5 min with 3 % (wt/vol) lead citrate. Visualization was done in a FEI Tecnai T12 Transmission Electron Microscope G2 Spirit Biotwin using Veleta camera Plus integrated software. Myofibrillar area, mitochondrial area and morphology and lipid droplet morphology were quantified in 6 images of representative myocytes (9.000–14.000× magnification) per sample using ImageJ software and averaged. Values were averaged per sample.

### 2.5 LV tissue proteomics

Proteomics was performed on LV tissue homogenates using in-gel digestion and nanoscale liquid chromatography coupled to tandem mass spectrometry. A detailed description is provided in the Supplementary files. Proteomics data are available via the ProteomeXchange Consortium via the PRIDE partner repository with the dataset identifier PXD043345.

### 2.6 LV tissue metabolomics and lipidomics

Metabolomics and lipidomics were performed using snap-frozen LV tissue as previously described [31,32]. A detailed description is provided in the Supplementary files. Metabolomics and lipidomics data are available via MetaboLights database under accession no. MTBLS7954.

### 2.7 Protein analysis by Western blot

Proteomics LV tissue homogenates were used. Proteins (30 μg per sample) were separated on a precast BioRad Criterion TGX gel and transferred to polyvinylidene difluoride membrane. Site-specific antibodies directed against cMyBP-C (Santa Cruz, sc13718; 1:5000 in Tris-buffered saline +0.1 % Tween (TBS-T) + 5 % (w/vol) non-fat dry milk (NFDM)), phospho-Ser273-cMyBP-C (1:2500 in TBS-T + 5 % NFDM), phospho-Ser282-cMyBP-C (1:5000 in TBS-T + 5 % NFDM), phospho-Ser302-cMyBP-C (1:10000 in TBS-T + 5 % NFDM) and cMyBP-C (1:4000 in 1:1 (vol/vol) TBS-T:TBS Blocking Buffer (LI-COR) (all kindly provided by Prof. Sakthivel Sadayappan (Loyola University, Chicago, IL, USA)) and tropomyosin (Abcam, ab133292; 1:4000 in 1:1 (vol/vol) TBS-T:TBS Blocking Buffer (LI-COR)) were used to detect proteins. Visualization was done using an enhanced chemiluminescence detection kit (Amersham) and scanning with an Amersham Imager 600 or with an LI-COR near-infrared detection system. Quantification was done via densitometric analysis.

### 2.8 Statistical analysis

Echocardiography, respirometry, Western blot and transmission electron microscopy (TEM) data were analyzed by two-way ANOVA with Tukey's multiple comparisons test using GraphPad Prism v9 software. *P* < 0.05 was considered significant. Data are displayed as mean ± standard error of the mean. All metabolomics and lipidomics data were log2 transformed and analyzed using R-limma [33]. *P*-values were corrected for multiple testing via the Benjamini-Hochberg FDR procedure. A corrected P-value (i.e. Q-value) <0.1 was considered significant. Differences in cardiac cardiolipin and acylcarnitine profiles of WD-fed WT and HET mice were explored by combining both metabolomic and lipidomic data, which were log2 transformed and analyzed using Welch's *t*-test without correcting for multiple testing.

## 3 Results

### 3.1 WD-induced derailment of metabolic health causes cardiac hypertrophy and dysfunction only in HET mice independent of cMyBP-C haploinsufficiency

Serum metabolomics was performed to identify WD-induced systemic effects (complete list of metabolites shown in Supplementary Table 1). Systemic effects reflective of unfavorable metabolic health are listed in Table 1. Increased abundance of acylcarnitines, ketones, sugar-derived metabolites and uric acid in WD- versus NC-fed mice is consistent with peripheral insulin resistance and disrupted glucose homeostasis [34,35]. In line with the high butter content of the WD, we observed higher (mono-un)saturated fat and lower unsaturated fat levels in WD-fed than in NC-fed mice. Glycerol-3-phosphate was also higher in WD-fed relative to NC-fed mice, which has previously been implicated in liver dysfunction and kidney injury [36,37]. Elevated levels of the arachidonic acid-derived prostanoid species 13,14-dihydro PGF-1α in WD-fed mice may be indicative of WD-induced systemic inflammation [38]. We did not observe any significant differences in serum metabolite levels between WD-fed WT and HET mice.

**Table 1:** Effect of Western diet-feeding on metabolic health-related metabolites in serum of normal chow and Western diet-fed mice*.*

Class	Metabolite	Fold change	Diet effect Q-value
Carnitines	AC(12:1)	1.50	3.66E-02
	AC(18:0)	1.43	7.96E-02
	AC(18:1)	1.39	5.92E-03
	AC(22:4) 23-Acetoxysoladulcidine	1.38	3.28E-02
	AC(22:6)	1.65	1.29E-04
	L-Acetylcarnitine	0.63	4.47E-03
	l-Carnitine	0.63	3.89E-02
Ketones	2-Hydroxybutyric acid	2.06	3.77E-03
	(*R*)-3-Hydroxybutyric acid		
	(*S*)-3-Hydroxyisobutyric acid		
	(*R*)-3-Hydroxyisobutyric acid		
	3-Hydroxybutyric acid		
	(*S*)-3-Hydroxybutyric acid		
	4-Hydroxybutyric acid		
	2-Methyl-3-hydroxypropanoate		
Sugar derivatives	Galactonic acid	1.77	8.13E-02
	Gluconic acid		
	Gulonic acid		
	D-Glucuronic acid	1.56	9.71E-02
	Galacturonic acid		
	Iduronic acid		
	3-Dehydro-L-gulonate		
	5-Keto-D-gluconate		
	2-Keto-L-gluconate		
	(L-)Glyceric acid	1.38	2.19E-02
Purine derivatives	Uric acid	1.67	2.89E-04
Fatty acids	FA(16:0) TrimethylFA(13:0)	1.41	1.71E-02
	FA(16:1)	1.34	8.26E-02
	DihydroxyFA(18:2) EpoxyFA(18:1/0)	0.74	5.40E-02
	FA(18:2)	0.71	8.68E-02
Glycerol derivatives	Glycerol 3-phosphate	1.50	1.33E-03
	Beta-Glycerophosphoric acid		
Prostanoids	13,14-Dihydro PGF-1a	1.64	1.71E-02

Fold change indicates abundance in WD-fed versus NC-fed animals, regardless of genotype. Data were analyzed using R-limma and *p*-values were corrected for multiple testing via the Benjamini-Hochberg FDR procedure. AC indicates acylcarnitine; FA, fatty acid; PGF, prostaglandin F. *N* = 8 individual mouse serum samples per group.

**Fig. 2:**
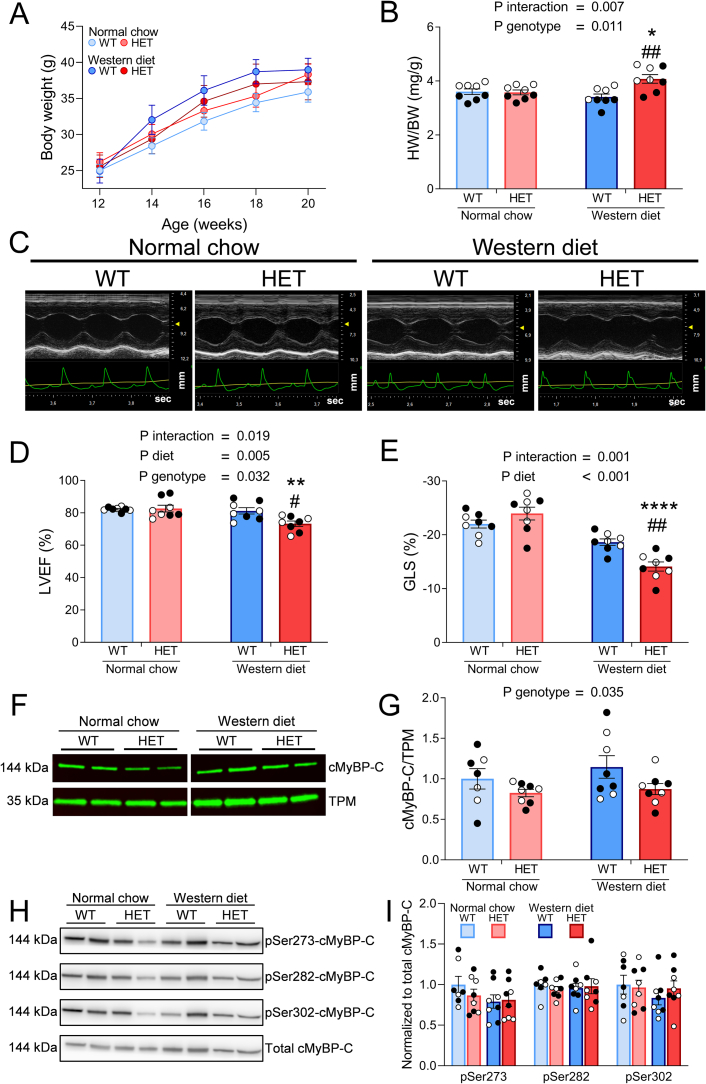
Western diet induces cardiac hypertrophy and cardiac impairment in heterozygous (HET) mice without affecting cardiac myosin binding protein-C (cMyBP-C) levels and phosphorylation status. *Twelve-week old wild-type (WT) and HET mice were fed a Western diet or normal chow for 8 weeks. Cardiac function was determined* via *echocardiography. Protein analyses were done* via *Western blot. A: Body weight over time; B: Heart weight (HW) to body weight (BW) ratio; C: Representative left ventricle (LV) short axis recordings showing impairment in Western diet-fed HET mice; D: LV ejection fraction (LVEF); E: Global longitudinal strain (GLS); F, G: Representative Western blot images and quantification of cMyBP-C and tropomyosin (TPM) levels. cMyBP-C levels were normalized to TPM. Data points represent average of 2 technical replicates per individual sample; H, I: Representative Western blot images and quantification of phospho-Ser-273-, phospho-Ser-282-, phospho-Ser-302- and total cMyBP-C levels. All phospho-cMyBP-C levels were normalized to total cMyBP-C levels. Data points represent 1 technical replicate per individual sample; A, B, D, E, G, I were analyzed* via 2-way ANOVA with Tukey's multiple comparisons test; *P *<* 0.05, ** P *<* 0.01, **** P *<* *0.0001 Western diet HET* vs *normal chow HET; #P* *<* *0.05, ##P* *<* *0.01 Western diet HET* vs *Western diet WT. N* *=* *8 individual mice per group in A-E; WT Normal chow N* *=* *7; HET Normal chow N* *=* *8; WT Western diet N* *=* *8; HET Western diet N* *=* *8 individual left ventricular cardiac tissue samples in F—I. Data are presented as mean ± SEM. White and black symbols indicate data from male and female mice, respectively.*

Body weight and cardiac parameters of NC- and WD-fed WT and HET mice are shown in Table 2 and Fig. 2. While the negative impact of WD-feeding on metabolic health was clear, WD-fed mice did not develop overt obesity (Fig. 2A). Diastolic function and LV longitudinal strain were moderately lower in WD-fed mice than in NC-fed mice. Strikingly, only HET WD-fed mice displayed a pronounced cardiac phenotype, which was evident from LV hypertrophy, reduced LV ejection fraction and impaired LV longitudinal strain (Table 2; Fig. 2B-E). These effects were similar in male and female mice. Since HET *Mybpc3* truncating mutations provoke disease by inducing cMyBP-C haploinsufficiency [6] we assessed whether this was more pronounced in WD-fed HET. Compared to WT mice cMyBP-C levels were 21 % lower in HET mice, but no effect of WD-feeding was observed (Fig. 2F-G; Supplementary Fig. 1). Hypophosphorylation of cMyBP-C, which may contribute to contractile impairment in cardiac disease [39], was also not observed in WD-fed HET mice (Fig. 2H-I; Supplementary Fig. 2). Taken together, these data suggest that cardiac disease in WD-fed HET mice is not driven by aggravated cMyBP-C haploinsufficiency or hypophosphorylation.

**Table 2:** Cardiac parameters of wild-type (WT) and heterozygous (HET) mice fed a Western diet or normal chow.

	Normal chow		Western diet		P-value		
	WT	HET	WT	HET	Interaction	Diet	Genotype
BW (g)	36.5 ± 1.1	38.4 ± 0.9	39.0 ± 1.6	37.3 ± 2.5	0.28	0.66	0.93
HW (mg)	131.6 ± 6.6	137.8 ± 6.3	133.3 ± 8.4	152 ± 11.8	0.47	0.36	0.16
HW/BW (mg/g)	3.6 ± 0.1	3.6 ± 0.1	3.4 ± 0.1	4.1 ± 0.2	**<0.01**	0.21	**<0.05**
Heart rate (bpm)	544.1 ± 5.8	551.3 ± 8.2	535.2 ± 10.1	563.6 ± 11.1	0.25	0.85	0.06
EF (%)	82.2 ± 0.5	82.6 ± 2.1	81.2 ± 1.9	73.3 ± 1.7	**<0.05**	**<0.01**	**<0.05**
E/A	1.4 ± 0.1	1.5 ± 0.1	1.2 ± 0.1	1.2 ± 0.1	0.68	**<0.05**	0.28
IVRT (ms)	12.8 ± 1.4	14.4 ± 1.0	15.7 ± 0.7	14.5 ± 0.4	0.14	0.11	0.81
GLS (%)	−22.0 ± 0.7	−24.0 ± 1.2	−18.6 ± 0.6	−14.1 ± 0.9	**<0.01**	**<0.001**	0.15
LVAW (mm)	1.23 ± 0.07	1.21 ± 0.07	1.27 ± 0.06	1.23 ± 0.06	0.73	0.76	0.75
LVPW (mm)	0.95 ± 0.07	1.07 ± 0.09	0.89 ± 0.05	0.87 ± 0.04	0.29	0.06	0.46
LVID (mm)	3.29 ± 0.10	3.17 ± 0.07	3.33 ± 0.11	3.36 ± 0.16	0.52	0.31	0.72

Data are presented as mean ± SEM. BW indicates body weight; HW, heart weight; EF, Ejection fraction; IVRT, isovolumetric relaxation time; GLS, global longitudinal strain; LVAW, end-diastolic left ventricular anterior wall thickness; LVPW, end-diastolic left ventricular posterior wall thickness; LVID, end-diastolic left ventricular internal diameter. *N* = 8 mice per group. All data were analyzed via 2-way ANOVA. P < 0.05 was considered significant.

### 3.2 WD suppresses mitochondrial respiration despite increased mitochondrial abundance

**Fig. 3:**
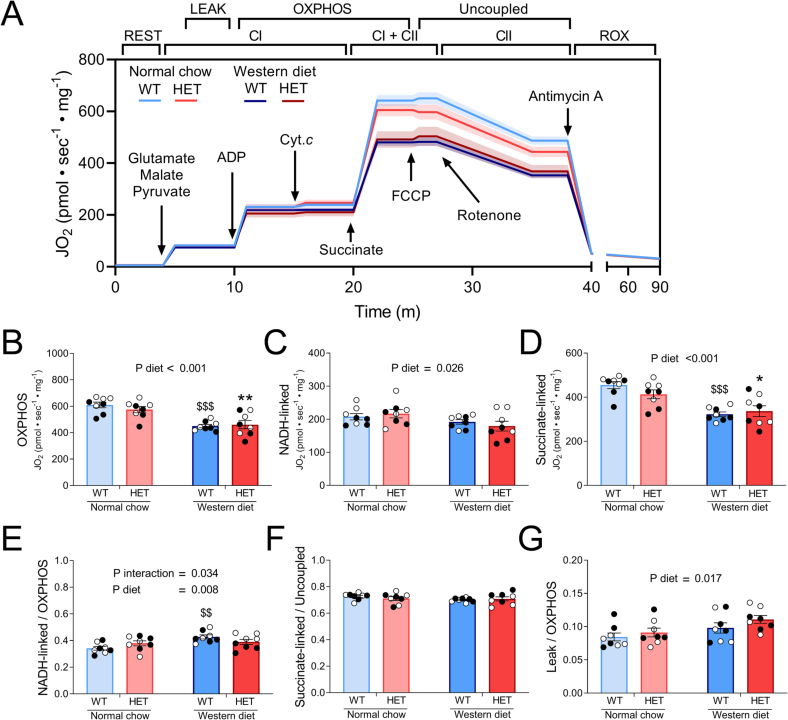
Western diet dampens mitochondrial respiration in permeabilized left ventricular tissue fibers of wild-type (WT) and heterozygous (HET) mice. *A: Averaged traces ±* *SEM of WT and HET mice fed a Western diet or normal chow; B: Total oxidative phosphorylation (OXPHOS) capacity; C: NADH-linked complex I respiration; D: Succinate-linked complex II respiration; E: NADH-linked respiration normalized to total OXPHOS capacity; F: Succinate-linked respiration normalized to uncoupled respiration; G: Leak respiration normalized to total OXPHOS capacity B-G were analyzed* via *2-way ANOVA; $$P* *<* *0.01, $$$P* *<* *0.001 Western diet WT* vs Normal chow WT; *P *<* 0.05; **P *<* *0.01 Western diet HET* vs *normal chow HET. Abbreviations: CI, complex I; CII, complex II; ROX; residual oxygen consumption; ADP, adenosine diphosphate; FCCP, carbonyl cyanide-p-trifluoro-methoxyphenylhydrazone. Data points represent average of 2 technical replicates. N* *=* *8 individual left ventricular cardiac tissue samples per group. Data are presented as mean ± SEM. White and black symbols indicate data from male and female mice, respectively.*

We measured mitochondrial respiration in permeabilized LV fibers to characterize alterations in mitochondrial oxidative phosphorylation (OXPHOS). Averaged traces of each experimental group are shown in Fig. 3A. WD-feeding reduced total OXPHOS capacity irrespective of genotype (23 % lower in WD-fed versus NC-fed mice; Fig. 3B). Both NADH-linked complex I (Fig. 3C) and succinate-linked complex II (Fig. 3D) respiration were depressed upon WD-feeding without significant difference between genotypes. The WD-mediated drop in succinate-linked respiration was larger than the decrease in NADH-linked respiration (24 % and 13 %, respectively). Since in particular succinate-driven respiration was markedly lower in WD-fed mice, NADH-linked complex I respiration expressed as fraction of total OXPHOS capacity was significantly higher upon WD-feeding in WT, though not in HET mice (Fig. 3E). This suggests that WD-feeding affected NADH- and succinate-driven respiration more proportionally in HET than in WT mice. Succinate-linked respiration expressed as proportion of uncoupled respiration did not display a significant interaction, diet or genotype effect (Fig. 3F). Independent of genotype, leak respiration expressed as fraction of total OXPHOS capacity was 19 % higher by WD-feeding (Fig. 3G). Overall, these data suggest a prominent effect of WD-feeding on mitochondrial function. Despite cardiac hypertrophy and dysfunction HET WD-fed mice did not show overt mitochondrial dysfunction compared to WT WD-fed mice.

**Fig. 4:**
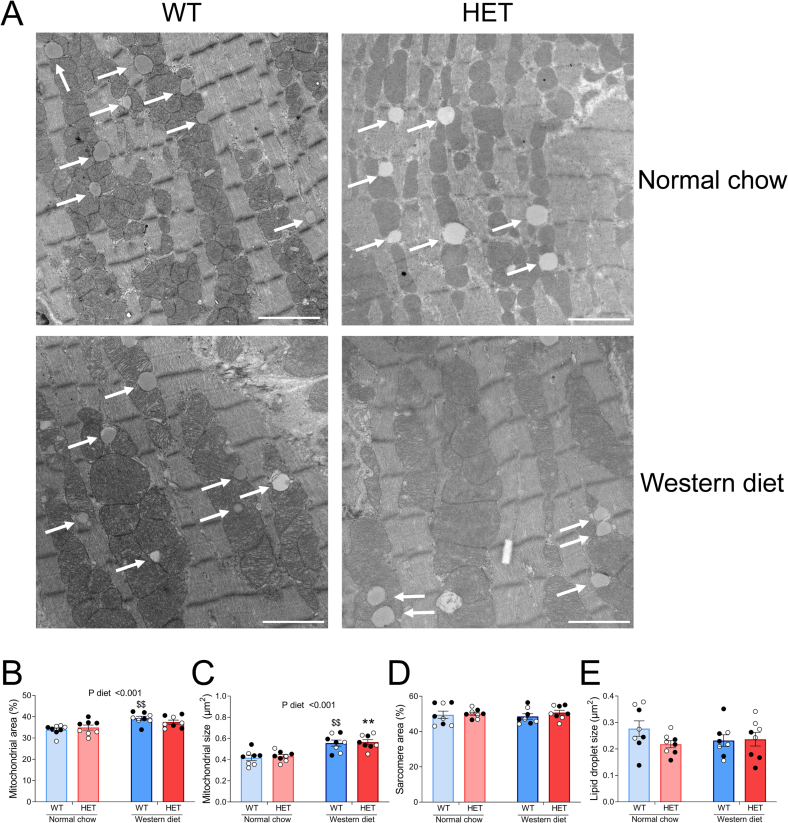
Increased mitochondrial abundance in Western diet-fed mice. *A: Representative transmission electron microscopy images of left ventricular tissue of Western diet or normal chow-fed wild-type (WT) and heterozygous (HET) mice. White arrows indicate lipid droplets. Scale bar indicates 2* μm*; B: Average mitochondrial area; C: Average individual mitochondrial size; D: Average sarcomere area; E: Average individual lipid droplet size. B-E were analyzed* via *2-way ANOVA; $$P* *<* *0.01 Western diet WT* vs Normal chow WT; **P *<* *0.01 Western diet HET* vs *Normal chow HET. Data points represent average of 6 images per individual left ventricular cardiac tissue sample. N* *=* *8 mice per group. Data are presented as mean ± SEM. White and black symbols indicate data from male and female mice, respectively.*

We used TEM to assess whether altered mitochondrial function was associated with changes in mitochondrial ultrastructure. Representative images are shown in Fig. 4A. We did not observe any qualitative changes in cristae structure between groups. Mitochondrial area (Fig. 4B) and average individual mitochondrial size (Fig. 4C) were 12 % and 32 % higher, respectively, in WD-fed animals regardless of genotype. Thus, these data suggest that the WD-induced dampening of mitochondrial function was not accompanied by mitochondrial loss or damage, but rather an increase in mitochondrial mass. This in turn did not appear to be linked to imbalance of mitochondrial fusion and fission, as markers of these processes (OPA1, MFN2, DRP1, FIS1, MFF) were not altered by WD-feeding (evaluated via proteomics; Supplementary Fig. 3). Total sarcomere area, assessed by TEM, was not altered in any experimental group (Fig. 4D), indicating contractile impairment in WD-fed HET mice cannot be attributed to loss of contractile machinery. No differences were observed in individual lipid droplet size (Fig. 4E), suggesting cardiac fat storage in lipid droplets did not appear to be increased despite systemic lipid overload and peripheral insulin resistance in WD-fed mice (Table 1).

### 3.3 WD induces metabolic shift towards fatty acid oxidation, which is blunted in HET compared to WT mice

**Fig. 5:**
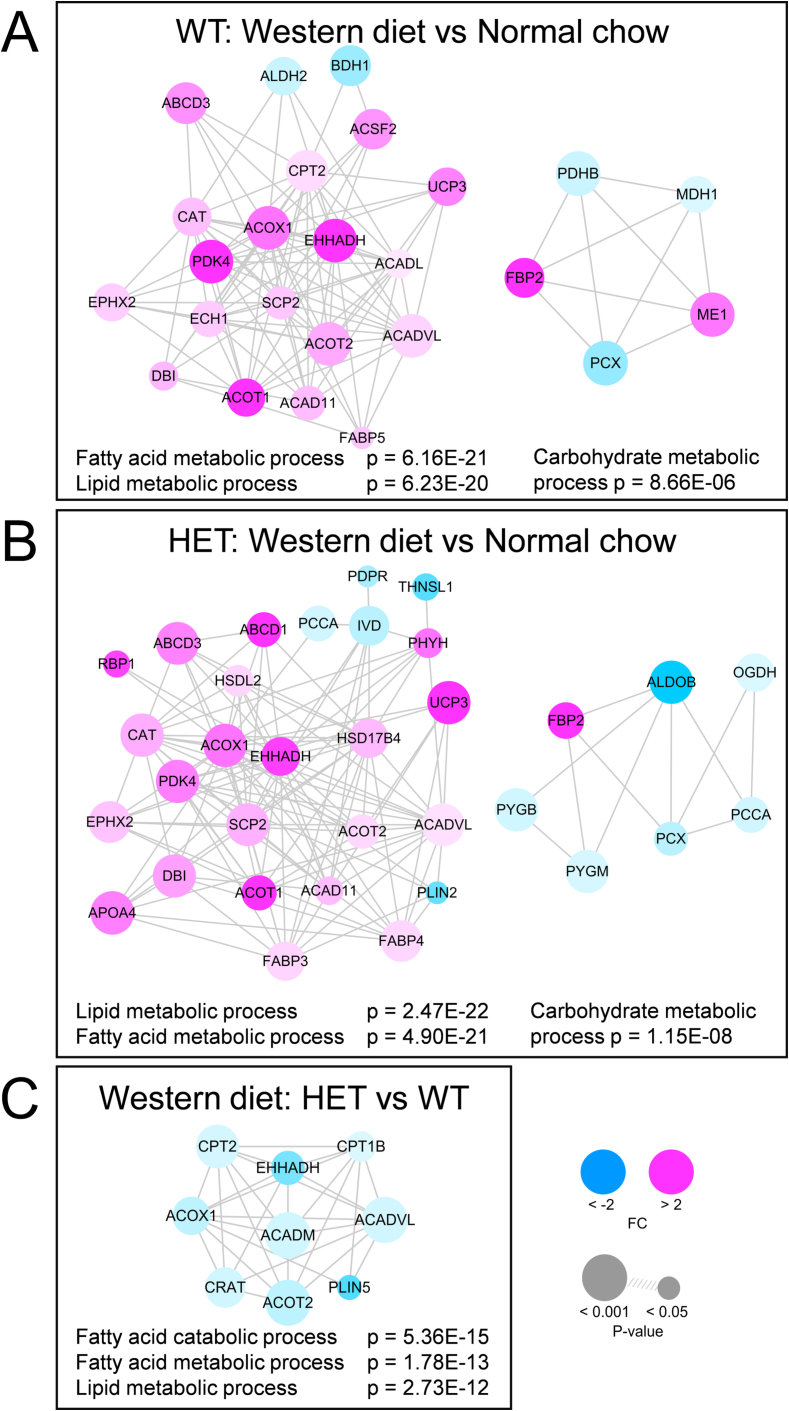
Western diet alters expression of proteins involved in fatty acid and carbohydrate metabolism in left ventricular tissue. Clusters of interacting proteins that were significantly different and corresponding gene ontology terms are shown for the group comparisons Western diet wild-type (WT) vs normal chow WT (A); Western diet heterozygous (HET) vs normal chow HET (B); Western diet HET vs Western diet WT (C). In both WT and HET mice Western diet induced expression of fatty acid/lipid metabolism-related proteins (A, B), which was partially blunted in Western diet-fed HET vs WT mice (C). FC indicates fold change. Color and size of protein nodes indicate FC between groups and significance of these changes, respectively. WT Normal chow *N* = 7; HET Normal chow N = 8; WT Western diet N = 8; HET Western diet N = 8 individual left ventricular cardiac tissue samples. Protein clusters were generated using ClusterONE and *p*-values were derived from Gene Ontology analysis using the BiNGO plug-in in Cytoscape [57,58].

We performed proteomics on LV tissue to compare differences in protein levels between groups. An overview of group comparisons and corresponding numbers of differently expressed proteins is provided in Supplementary Table 2. WT and HET WD-fed mice displayed a considerable amount of overlap (≈35 %) in differently abundant proteins compared to NC-fed mice (Supplementary Fig. 4). To determine which cellular processes were altered in each group comparison, we performed GO analysis on clusters of interacting proteins (according to STRING database [40]). In both WT and HET mice the most prominent cluster of proteins altered in WD-fed compared to NC-fed animals was associated with metabolism of fatty acids and lipids (Fig. 5A-B, Supplementary Table 3). Specifically, proteins involved in fatty acid/lipid binding (APOA4, SCP2, DBI in WT and HET; APOA1, APOE, FABP5 in WT; FABP3, FABP4 in HET) and oxidation of fatty acids in peroxisomes (ABCD3, ACOX1, EHHADH in WT and HET; ABCD1, HSD17B4, HSDL2, PHYH in HET) and mitochondria (ACADVL, ACAD11 in WT and HET; CPT2, ACSF2, ACADL, ECH1 in WT) were more abundant in WD-fed versus NC-fed mice. These findings imply that fatty acids were used more as substrate for energy metabolism in WD-fed compared to NC-fed mice. In both WT and HET a cluster of proteins involved in carbohydrate metabolism was significantly altered by WD-feeding (Fig. 5A-B). This was characterized by lower abundance of proteins involved in tricarboxylic acid (TCA) cycle input via pyruvate (PCX in WT and HET; PDHB in WT; ALDOB in HET) and TCA cycle-associated enzymes (MDH1 in WT; OGDH in HET). Higher levels of PDK4 (negative regulator of pyruvate dehydrogenase) in both WD-fed groups is in line with reduced pyruvate oxidation and upregulation of fatty acid oxidation [41]. Among WD-fed mice, HET mice displayed lower abundance of a cluster of proteins involved in fatty acid catabolism, specifically via peroxisomal (ACOX1, EHHADH) and mitochondrial (CPT1B, CPT2, CRAT, ACADVL, ACADM) fatty acid oxidation compared to WT mice (Fig. 5C). Collectively, these findings suggest that WD-feeding in both genotypes lowered abundance of proteins involved in oxidative glucose metabolism and upregulated levels of fatty acid oxidation proteins. However, in WD-fed HET mice upregulation of fatty acid oxidation proteins appeared to be blunted compared to WD-fed WT mice.

**Fig. 6:**
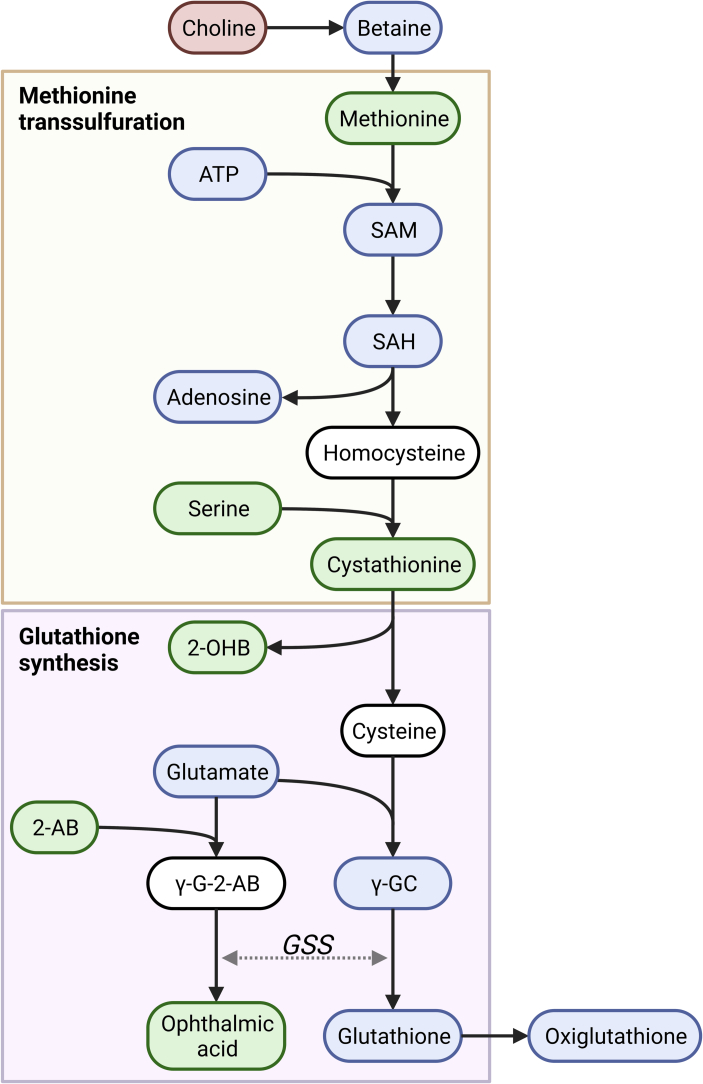
Alterations in levels of metabolites involved in methionine transsulfuration and glutathione synthesis in Western diet-fed versus normal chow-fed mice irrespective of genotype. Red, green and blue metabolites indicate significantly lower, significantly higher and unchanged abundance respectively in Western diet-fed versus normal chow-fed mice according to R-limma-derived Q-values. White metabolites were not detected. Methionine, serine and cystathionine were elevated while choline was reduced in WD-fed mice compared to NC-fed mice. Cysteine conversion from cystathionine directed towards glutathione synthesis yields 2-hydroxybutyric acid, which was increased in WD-fed mice versus NC-fed mice. Glutathione is subject to rapid conjugated and was not altered. Ophthalmic acid and 2-aminobutyric acid, which are implicated in glutathione consumption and homeostasis [42,43] were elevated in WD-fed mice versus NC-fed mice. ATP indicates adenosine triphosphate; SAM, S-adenosyl methionine; SAH, S-adenosyl homocysteine; 2-OHB, 2-hydroxybutyrate; 2-AB, 2-aminobutyrate; γ-G-2-AB, γ-glutamyl-2-aminobutyrate; γ-GC, γ-glutamylcysteine; GSS, glutathione synthetase. WT Normal chow N = 7; HET Normal chow N = 8; WT Western diet N = 8; HET Western diet N = 8 individual left ventricular cardiac tissue samples. Created with BioRender.com (For interpretation of the references to color in this figure legend, the reader is referred to the web version of this article.)

Semi-targeted polar metabolomics and lipidomics were performed to characterize changes in metabolic and lipid profiles in LV tissue. Metabolites that displayed a significant diet effect (Q < 0.1) are shown in Table 3; no significant genotype or interaction effects were found. Several metabolites involved in cysteine synthesis via the methionine transsulfuration pathway (labeled yellow) were increased in WD-fed mice compared to NC-fed mice (methionine, serine, cystathionine). This was accompanied by a reduction in choline, which may fuel methionine synthesis. 2-Hydroxybutyric acid, which is formed when cystathionine is converted to cysteine that is directed towards glutathione synthesis, was increased in WD-fed mice, hinting towards increased glutathione production. We did not observe higher glutathione levels in WD-fed vs NC-fed mice, which may be explained by increased consumption via conjugation. Ophthalmic acid, which is generated by the same enzyme (glutathione synthetase) that catalyzes the final step of glutathione synthesis and is considered a marker of glutathione consumption [42], was increased however in WD-fed versus NC-fed mice. 2-Aminobutyric acid, which fuels ophthalmic acid formation and has been linked to maintaining glutathione homeostasis [43], was also elevated in WD-fed compared to NC-fed mice. Combined, these data may suggest that in WD-fed mice methionine transsulfuration-mediated glutathione synthesis was increased (summarized in Fig. 6).

**Table 3:**
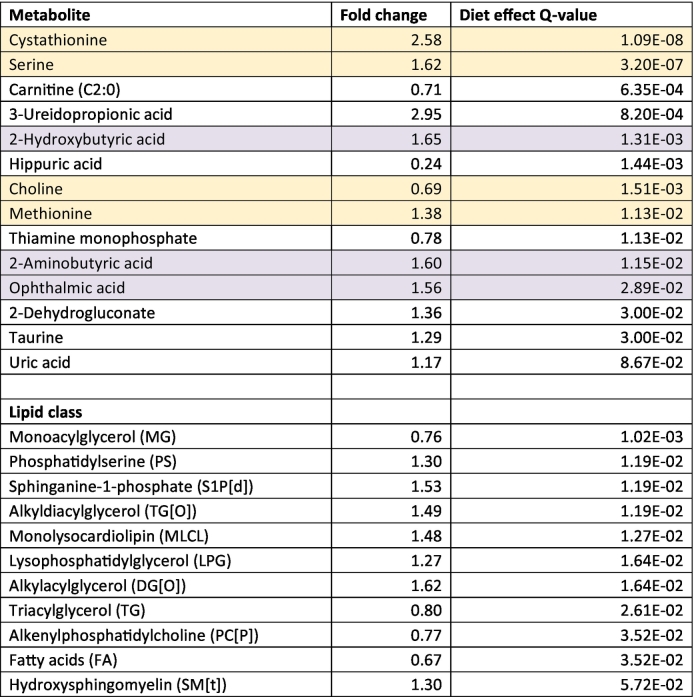
Effect of Western diet-feeding on metabolomic and lipidomic profile in cardiac tissue of normal chow and Western diet-fed mice.

Fold change indicates abundance in WD versus NC-fed animals, regardless of genotype. Data were analyzed using R-limma and p-values were corrected for multiple testing via the Benjamini-Hochberg FDR procedure. WT Normal chow N = 7; HET Normal chow N = 8; WT Western diet N = 8; HET Western diet *N* = 8 individual left ventricular cardiac tissue samples. Created with BioRender.com

**Fig. 7:**
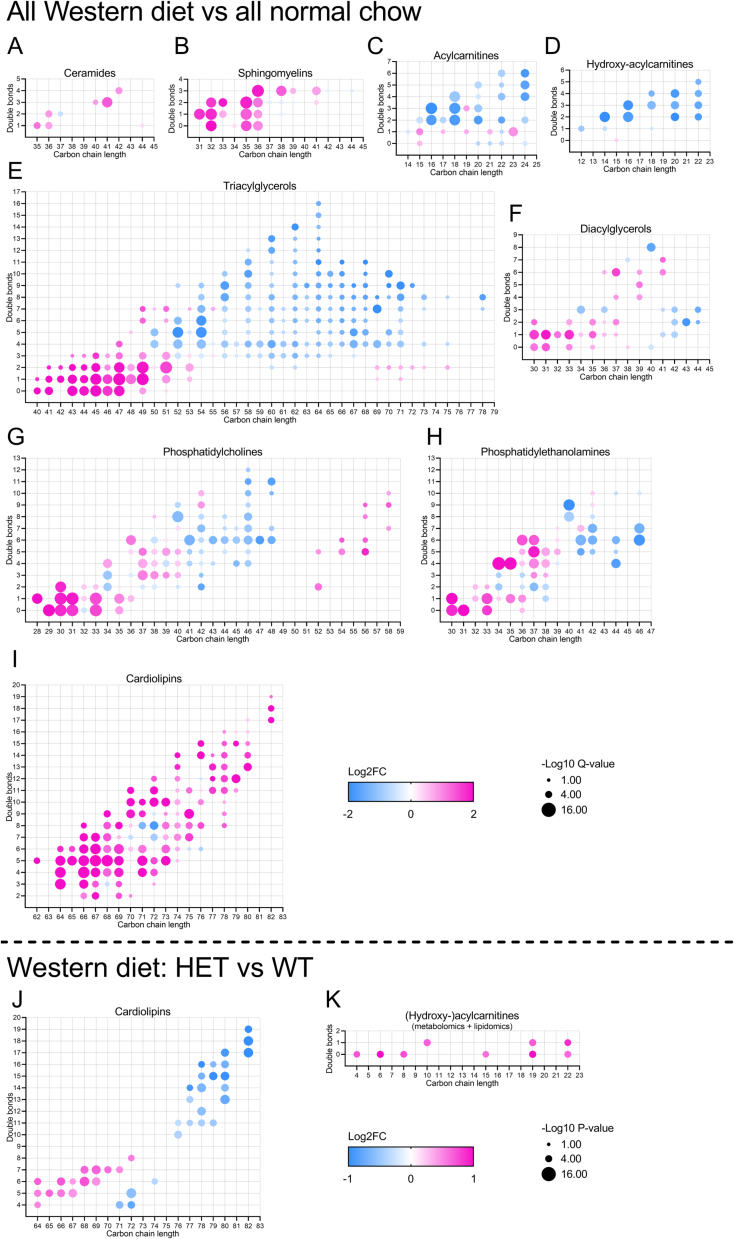
Western diet elevates levels of toxic lipids and alters composition of lipid storage pools and phospholipids independently of genotype; Western diet alters cardiolipin profile and raises levels of multiple acylcarnitines in heterozygous (HET) versus wild-type (WT) mice. Significantly different lipid species in left ventricular cardiac tissue are displayed in dot plots, in which each dot represents a significantly altered lipid species. FC indicates fold change. Vertical axes indicate double bond number and horizontal axes indicate carbon chain length of lipid species. Color and size of dots indicate FC between groups and significance of these changes, respectively. (A-I) show significant differences at the level of diet after correcting for multiple testing (analyzed via R-limma and corrected via the Benjamini-Hochberg procedure); (J-K) show differences between Western diet-fed HET and WT mice (analyzed via Welch's *t*-test without multiple testing correction). (K) displays combined data of (hydroxy-)acylcarnitines from metabolomics and lipidomics analysis. WT Normal chow N = 7; HET Normal chow N = 8; WT Western diet N = 8; HET Western diet N = 8 individual left ventricular cardiac tissue samples.

Lipid classes that were different at the level of diet are displayed in Table 3 and major diet-induced changes in lipid composition within lipid classes are summarized in Fig. 7. Levels of free fatty acids and mono/triacylglycerols were lower in hearts of WD-fed compared to NC-fed mice (Table 3), suggesting that free fatty acids did not accumulate and were not stored more in lipid depots, despite increased fatty acid supply. WD-fed mouse hearts displayed higher total amounts of sphinganine-1-phosphate and hydroxysphingomyelin (Table 3) and increased levels of multiple ceramide and sphingomyelin species (Fig. 7A-B), suggestive of lipotoxicity. In WD-fed versus NC-fed mice numerous long-chain polyunsaturated (hydroxy-)acylcarnitines were less abundant while several (monoun)saturated (hydroxy-)acylcarnitines were slightly elevated (Fig. 7C-D). Triacylglycerols, diacylglycerols, phosphatidylcholines and phosphatidylethanolamines generally contained shorter and more saturated acyl chains in WD-fed versus NC-fed mice (Fig. 7E-H). Whereas total cardiolipin levels were unchanged, we observed striking WD-induced remodeling of the cardiolipin pool, in which the major species (tetralinoleoyl-cardiolipin; CL72:8) was lowered while levels of 101 other species were higher in WD-fed compared to NC-fed mice (Fig. 7I). We did not observe any genotype or interaction effects for any lipid. To explore possible disease-specific changes we compared WD-fed HET mice to WD-fed WT mice without correcting for multiple testing, and screened for patterns of differently abundant lipids within lipid classes. This revealed a shift in cardiolipin composition to shorter and more saturated acyl chains in WD-fed HET vs WT mice (Fig. 7J). Since WD-fed HET mice displayed lower abundance of fatty acid oxidation enzymes compared to WD-fed WT mice (Fig. 5C), we combined metabolomics and lipidomics-derived data of (hydroxyl-)acylcarnitines and compared levels between these groups of mice (Fig. 7K). We observed elevation of 9 acylcarnitine species in WD-fed HET versus WT mice, indeed suggesting the WD-induced increase in fatty acid oxidation capacity may be dampened in HET mice.

## 4 Discussion

**Fig. 8:**
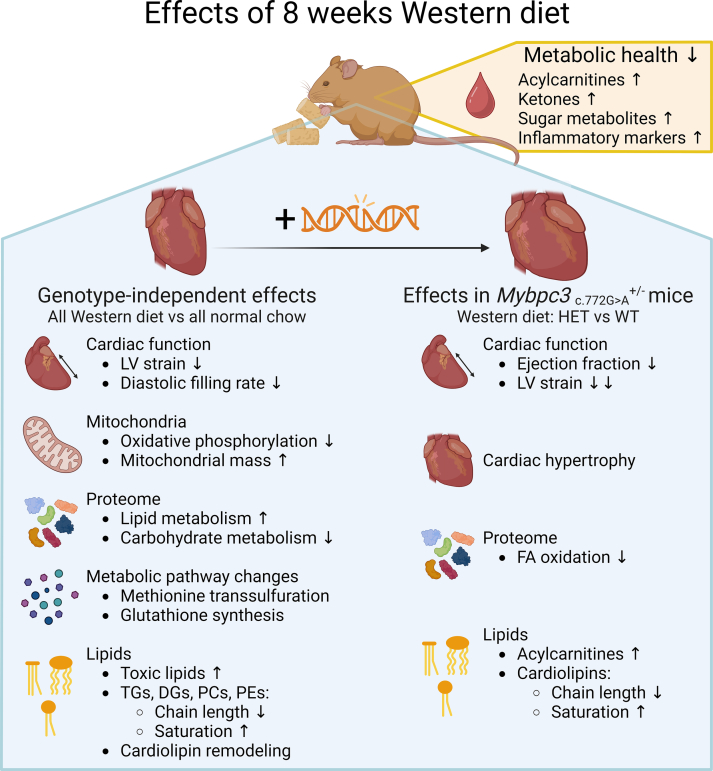
Summary of the main findings in this study. Eight weeks of Western diet-feeding induced an unfavorable metabolic health profile in serum metabolites in both wild-type and heterozygous mice. Western diet-fed mice of both genotypes displayed a wide array of cardiac alterations compared to normal chow-fed mice: left ventricular (LV) strain and diastolic filling were mildly impaired; mitochondrial oxidative phosphorylation was depressed while mitochondrial mass increased; numerous proteins involved in lipid metabolism were more abundant whereas carbohydrate metabolism-related proteins were depressed; the methionine transsulfuration and glutathione synthesis pathways were altered and major lipid changes included formation of toxic lipids and remodeling of fat storage pools (triacylglycerol, TG; diacylglycerol, DG), phospholipids (phosphatidylcholine, PC; phosphatidylethanolamine, PE) and cardiolipin. Only heterozygous mice developed a cardiac disease phenotype upon Western diet-feeding. Additional Western diet-induced effects observed in these mice included: cardiac hypertrophy, depressed LV ejection fraction and a further reduction in LV strain; blunted upregulation of fatty acid (FA) oxidation proteins and accumulation of acylcarnitines and a shift in cardiolipin composition. Created with BioRender.com

In this study, we demonstrated that WD-feeding triggered cardiac dysfunction and hypertrophy in otherwise phenotype-negative HET *Mybpc3*-targeted knock-in mice. An overview of the results of our study is provided in Fig. 8. Our main findings are that WD-feeding independently of genotype gave rise to 1) systemic alterations reflecting unfavorable metabolic health; 2) subtle changes in cardiac function; 3) reduced mitochondrial oxidative phosphorylation in spite of increased mitochondrial mass; 4) increased abundance of proteins involved in lipid metabolism; 5) changes in the methionine transsulfuration and glutathione synthesis pathways and 6) elevated levels of toxic lipid species and remodeling of fat storage pools and phospholipids. In HET versus WT mice, WD-feeding induced 1) cardiac dysfunction and hypertrophy; 2) a blunted upregulation of fatty acid oxidation proteins and 3) higher abundance of acylcarnitines and shortening and desaturation of the cardiolipin pool. Patients with HCM are typically HET for a genetic variant and display dramatic variation in disease penetrance and outcome [14,15]. Recent reports suggest unfavorable metabolic health may sensitize disease in a substantial number of individuals carrying a HET pathogenic gene variant [[19], [20], [21], [22], [23], [24], [25], [26]], suggesting a multifactorial disease cause in this population. Here we demonstrated that perturbed systemic metabolic health can indeed trigger disease in myocardium harboring a heterozygous sarcomere mutation, and thus managed to recapitulate a complex disease etiology in mice. This represents a major advancement from homozygous mouse models displaying extreme disease phenotypes towards more human-like HET mouse models of HCM showing milder phenotypes resulting from both genetic and environmental disease factors. Our approach may serve as a valuable model to study how systemic metabolic derailment predisposes myocardium harboring an HCM-linked mutation to disease development. Approximately 40 % of patients with HCM suffer from obesity-associated perturbations of metabolic health [[44], [45], [46]]. Understanding mechanisms through which perturbed metabolic health associated with obesity aggravates disease also at manifest stages of HCM will aid in developing therapies tailored specifically to this patient population.

### 4.1 Loss of metabolic flexibility in HET mice

Eight weeks of WD-feeding brought about major metabolic changes in the heart in both WT and HET mice. Maximal oxidative phosphorylation capacity was depressed and simultaneously a shift away from glucose oxidation towards relatively less oxygen-efficient fatty acid oxidation appeared to occur according to our proteomic findings, which may affect bioenergetic homeostasis. This shift was dampened in HET mice, which was reflected by increased levels of multiple acylcarnitine species. Cardiomyocytes of HET mice were previously described to display increased myofilament calcium sensitivity [16] and haploinsufficiency of cMyBP-C has been associated with increased energetic cost of cardiac contraction and impaired myosin super-relaxation [47,48], all of which representing mechanisms that have been linked to increased ATP consumption [49,50]. Truncating mutations in *Mybpc3* may slow down cMyBP-C turnover [51]. Thus, cMyBP-C in HET hearts may be more prone to metabolic stress-induced post-translational modifications that further affect myofilament efficiency [52], warranting further research. We propose that due to an increase in ATP demand hearts of mutant mice cannot cope with oxidative phosphorylation impairment and rely more on fatty acid oxidation under unfavorable metabolic health conditions, triggering development of overt cardiac dysfunction and hypertrophy. Inability to sufficiently oxidize fatty acids as energetic fuel as an early pathological change associated with cardiac remodeling is in line with recent findings that the degree of fatty acid oxidation impairment in myectomy tissue from patients with obstructive HCM was linked to the severity of septal hypertrophy [30]. We found no evidence for a relative upshift of (oxidative) glucose metabolism in WD-fed HET versus WT mice in our proteomics screening, which is to be expected in the case of decreased myocardial insulin sensitivity and impaired glucose uptake. However, we also found no depletion of ATP and TCA-cycle intermediates in WD-fed HET mice, suggesting alternative substrates were used to maintain energy production. Thus in future studies it would be interesting to unravel whether these hearts relied more on fuels such as ketones and amino acids. Taken together, our findings suggest the presence of a pathogenic gene variant in combination with derailed metabolic health leads to complex metabolic remodeling in the heart, warranting further study.

### 4.2 Limitations

We acknowledge several limitations in our study. First, we analyzed serum samples via metabolomics to characterize systemic changes induced by WD-feeding. We recognize this provides indirect insight into dietary fat intake, glucose tolerance and insulin resistance in WD-fed mice. Nonetheless, the systemic response to WD-feeding was clearly similar in WT and HET mice. Second, we did not functionally evaluate fatty acid oxidation capacity in cardiac tissue. However, the combination of a blunted increase in fatty acid oxidation proteins and accumulation of acylcarnitines in WD-fed HET vs WT mice strongly suggests fatty acid oxidation activity is dampened in the former versus the latter group. Third, the cardiac phenotype we observed in WD-fed HET mice was relatively mild and may become more pronounced when mice are exposed to WD-feeding for a longer period. The approach presented here thus captured especially early disease events, which are of particular interest in identifying therapeutic targets to halt disease progression. Fourth, we used both male and female mice, which may increase data variability and obscure some results. Nevertheless, by combining both sexes we managed to identify effects that are shared in male and female mice.

## 5 Conclusion

Here, we managed to induce cardiac dysfunction and hypertrophy in otherwise phenotype-negative HET *Mybpc3*-targeted knock-in mice using WD-feeding, mimicking a multi-hit disease etiology that is observed in a substantial part of the HCM patient population. Compromised fatty acid oxidation capacity represented the major pathological change in diseased mice. While a shift from fatty acid oxidation to more oxygen-efficient glucose oxidation has been proposed to be beneficial during cardiac stress [53], inhibition of fatty acid oxidation was found to exacerbate cardiac hypertrophy caused by pressure overload [54,55], suggesting that maintaining fatty acid oxidation capacity exerts cardioprotective effects under stress conditions [56]. Thus, based on our study, it would be interesting to study if boosting cardiac fatty acid oxidation in our and other models of HCM will prevent or rather aggravate disease development due to increased oxygen demand.

## Declaration of competing interest

All authors report no conflict of interest.

## Data Availability

The data underlying this article will be shared on reasonable request to the corresponding author.

## Appendix A Supplementary data

Supplementary material Image 1
