# Supplementary material for: Western diet triggers cardiac dysfunction in heterozygous Mybpc3-targeted knock-in mice: A two-hit model of hypertrophic cardiomyopathy
Source: J Mol Cell Cardiol Plus. 2023 Sep 19;6:100050. doi: 10.1016/j.jmccpl.2023.100050 (PMC11708371; doi:10.1016/j.jmccpl.2023.100050)
Supplement: Supplementary file 1 — Supplementary material [file mmc1.docx]

# SUPPLEMENTARY FILES

# Supplementary methods

## Mitochondrial function

Fresh left ventricular (LV) fiber bundles (≈2 mg) were gently separated in ice-cold preservation solution (BIOPS solution), containing (in mM) ­K_2_-ethyleneglycoltetraacetic acid (EGTA; 7.2), CaK_2_EGTA (2.8), ATP (5.8), MgCl_2_ (6.6), taurine (20), phosphocreatine (15), imidazole (20), dithiothreitol (0.5) and 2-(N-morpholino)ethanesulfonic acid (50); pH 7.1 adjusted with KOH, and subsequently permeabilized in ice-cold BIOPS solution containing 50 µg/mL saponin for 25 min. Next, fiber bundles were washed twice for 10 min in ice-cold mitochondrial respiration medium (MiR05), containing (in mM) EGTA (0.5), MgCl_2_ (3), potassium lactobionate (60), taurine (20), KH_2_PO_4_ (10) HEPES (20), sucrose (110) and 1 g/L fatty acid free bovine serum albumin, pH 7.1 adjusted with KOH, rapidly blotted dry, weighed and inserted into a high-resolution respirometer (Oxygraph-2k; Oroboros Instruments). Leak respiration was measured using 10 mM sodium glutamate, 2 mM sodium malate and 5 mM sodium pyruvate, providing electron input into complex I via NADH. Maximal NADH-linked respiration was measured upon addition of 5 mM ADP. Cytochrome-*c* (10 µM) was injected to evaluate outer mitochondrial membrane integrity. Total OXPHOS capacity, with maximum electron input through complexes I and II was assessed after adding 10 mM succinate. Carbonyl cyanide p-trifluoro-methoxyphenyl hydrazone (FCCP) was titrated in 0.05 μM steps to uncouple respiration from complex V, thus measuring excess capacity of the electron transferring complexes (I-IV). Rotenone (0.5 µM) was added to block complex I and assess succinate-linked respiration through complex II. Antimycin-A (2.5 µM) was injected to fully block mitochondrial oxygen consumption and measure residual oxygen consumption, which was subtracted from all values as background. Measurements were carried out at 37 °C under oxygen levels above 300 µM throughout the experiment to avoid oxygen supply limitations.

## LV tissue proteomics

### Sample preparation

Frozen LV tissue was pulverized and homogenized using a glass tissue grinder in 40 µL reducing sample buffer (106 mM Tris-HCl, 141 mM Tris-base, 0.51 mM ethylenediaminetetraacetic acid, 0.22 mM Coomassie Brilliant Blue G250, 0.18 mM phenol red, 100 mM dithiothreitol, 2% (wt/vol) lithium dodecyl sulfate, 10% (vol/vol) glycerol) per mg of tissue. Proteins were denatured at 99 °C for 5 min, sonicated using a sonifier (Branson; 3x 10s pulses, 70% intensity) and heated again at 99 °C for 5 min. Samples were centrifuged at maximum speed for 10 min in a microcentrifuge. Supernatants were collected and stored at -80 °C.

### Protein separation

To separate proteins, 30 µL of homogenate per sample (circa 30 µg protein) was loaded on 4-12% precast NuPAGE Bis-Tris mini gels (Invitrogen) followed by electrophoresis at 200 V in NuPAGE MES SDS running buffer until the dye reached the bottom of the gel. Gels were fixed in fixing solution (50% ethanol, 3% phosphoric acid). Proteins were stained using 0.1% Coomassie Brilliant Blue G-250 solution (1 g/L Coomassie Brilliant Blue G-250 in 34% methanol, 3% phosphoric acid, 15% ammonium sulfate).

### In-gel digestion and nano-LC-MS/MS

Each gel lane was separated into 5 slices, each roughly containing an equal amount of protein. In-gel digestion was performed as previously described (1). Samples were measured by liquid chromatography-mass spectrometry (LC-MS) on individual gel slice extracts. Gel slice extracts were measured group-wise, starting with the top gel slice extracts with highest molecular weight range, and ending with the bottom gel slice extracts with lowest molecular weight range. Analysis was performed as previously described (2). Peptides were separated using an Ultimate 3000 Nano LC-MS/MS system (Dionex LC-Packings) equipped with a 40 cm × 75 μm ID fused-silica column custom packed with 1.9 μm,120 Å ReproSil Pur C18 aqua (Dr Maisch GMBH). Eluting peptides were ionized at a potential of +2 kV into a Q Exactive mass spectrometer (Thermo Fisher). MS/MS spectra were acquired at resolution 17500 (at m/z 200) in the orbitrap using an AGC target value of 1×10^6^ charges, a maxIT of 60 ms, and an underfill ratio of 0.1%. Dynamic exclusion was applied using a repeat count of 1 and an exclusion time of 30s.

### Data analysis

MS/MS spectra were searched against a Uniprot mouse reference proteome FASTA file (2018_02, canonical and isoforms, 25131 entries) supplemented with FASTA data for retention time standards (Biognosis iRT kit) using MaxQuant version 1.5.4.1. Beta-binominal statistics were used to evaluate differential protein expression between groups, after normalization on the sum of the counts for each sample (3). Proteins with a P-value below 0.05 were considered significantly differentially abundant. Proteins that were detected in <25% of the samples or had an average normalized count of <1.4 were excluded from further analysis. Proteins with an uncorrected P-value <0.05 were selected for pathway analysis. Protein networks were obtained using the STRING database (4) and visualized in Cytoscape (5). Protein clusters were generated using ClusterONE and gene ontology (GO) analysis was conducted via the BiNGO plug-in in Cytoscape (6, 7). Only protein clusters in which ≥ 70% of proteins shared one or more GO terms were considered meaningful and are shown. Venn diagram was generated using InteractiVenn (8).

## LV tissue metabolomics and lipidomics

### Sample preparation

Metabolomics and lipidomics were performed using snap-frozen LV tissue as previously described (9, 10), with minor adjustments. Briefly, in a 2 mL tube containing 3 mg of freeze-dried LV cardiac tissue, the following amounts of internal standard dissolved in water were added to each sample for metabolomics: adenosine-^15^N_5_-monophosphate (5 nmol), adenosine-^15^N_5_-triphosphate (5 nmol), D_4_-alanine (0.5 nmol), D_7_-arginine (0.5 nmol), D_3_-aspartic acid (0.5 nmol), D_3_-carnitine (0.5 nmol), D_4_-citric acid (0.5 nmol), ^13^C_1_-citrulline (0.5 nmol), ^13^C_6_-fructose-1,6-diphosphate (1 nmol), ^13^C_2_-glycine (5 nmol), guanosine-^15^N_5_-monophosphate (5 nmol), guanosine-^15^N_5_-triphosphate (5 nmol), ^13^C_6_-glucose (10 nmol), ^13^C_6_-glucose-6-phosphate (1 nmol), D_3_-glutamic acid (0.5 nmol), D_5_-glutamine (0.5 nmol), D_5_-glutathione (1 nmol), ^13^C_6_-isoleucine (0.5 nmol), D_3_-lactic acid (1 nmol), D_3_-leucine (0.5 nmol), D_4_-lysine (0.5 nmol), D_3_-methionine (0.5 nmol), D_6_-ornithine (0.5 nmol), D_5_-phenylalanine (0.5 nmol), D_7_-proline (0.5 nmol), ^13^C_3_-pyruvate (0.5 nmol), D_3_-serine (0.5 nmol), D_6_-succinic acid (0.5 nmol), D_4_-thymine (1 nmol), D_5_-tryptophan (0.5 nmol), D_4_-tyrosine (0.5 nmol), D_8_-valine (0.5 nmol). In the same 2 mL tube, the following amounts of internal standards dissolved in 1:1 (vol/vol) methanol:chloroform were added for lipidomics: Bis(monoacylglycero)phosphate: BMP(14:0)_2_ (0.2 nmol), Ceramide-1-phosphate: C1P (d18:1/12:0) (0.125 nmol), D_7_-Cholesteryl Ester: CE(16:0) (2.5 nmol), Ceramide: Cer(d18:1/12:0) (0.125 nmol), Ceramide: Cer(d18:1/25:0) (0.125 nmol), Cardiolipin: CL(14:0)_4_ (0.1 nmol), Diacylglycerol: DAG(14:0)_2_ (0.5 nmol), Glucose Ceramide: GlcCer(d18:1/12:0) (0.125 nmol), Lactose Ceramide: LacCer(d18:1/12:0) (0.125 nmol), Lysophosphatidicacid: LPA(14:0) (0.1 nmol), Lysophosphatidylcholine: LPC(14:0) (0.5 nmol), Lysophosphatidylethanolamine: LPE(14:0) (0.1 nmol), Lysophosphatidylglycerol: LPG(14:0) (0.02 nmol), Phosphatidic acid: PA(14:0)_2_ (0.5 nmol), Phosphatidylcholine: PC(14:0)_2_ (2 nmol), Phosphatidylethanolamine: PE(14:0)_2_ (0.5 nmol), Phosphatidylglycerol: PG(14:0)_2_ (0.1 nmol), Phosphatidylinositol: PI(8:0)_2_ (0.5 nmol), Phosphatidylserine: PS(14:0)_2_ (5 nmol), Sphinganine 1-phosphate: S1P(d17:0) (0.125 nmol), Sphinganine-1-phosphate: S1P(d17:1) (0.125 nmol), Ceramide phosphocholines: SM(d18:1/12:0) (2.125 nmol), Sphingosine: SPH(d17:0) (0.125 nmol), Sphingosine: SPH(d17:1) (0.125 nmol), Triacylglycerol: TAG(14:0)_3_ (0.5 nmol). Subsequently, solvents were added to achieve a total volume of 500 µL water, 500 µL methanol and 1 mL chloroform. Cardiac tissues were homogenized before the addition of chloroform using a Qiagen TissueLyser II for 5 min at 30 times/s with a 5 mm Qiagen Stainless Steel Bead in each tube. All samples were thoroughly mixed and centrifuged for 10 min at 14.000 rpm.

### Metabolomics

The top layer, containing the polar phase, was transferred to a clean 1.5 mL tube and dried using a vacuum concentrator at 60 °C. Dried samples were reconstituted in 100 µL 6:4 (vol/vol) methanol:water. Metabolites were analyzed using a Waters Acquity ultra-high performance liquid chromatography system coupled to a Bruker Impact II™ Ultra-High Resolution Qq-Time-Of-Flight mass spectrometer. Samples were kept at 12 °C during analysis and 5 µL of each sample was injected. Chromatographic separation was achieved using a Merck Millipore SeQuant ZIC-cHILIC column (PEEK 100 x 2.1 mm, 3 µm particle size). Column temperature was held at 30 °C. Mobile phase consisted of (A) 1:9 (vol/vol) acetonitrile:water and (B) 9:1 (vol/vol) acetonitrile:water, both containing 5 mM ammonium acetate. Using a flow rate of 0.25 mL/min, the LC gradient consisted of: Dwell at 100% Solvent B, 0-2 min; Ramp to 54% Solvent B at 13.5 min; Ramp to 0% Solvent B at 13.51 min; Dwell at 0% Solvent B, 13.51-19 min; Ramp to 100% B at 19.01 min; Dwell at 100% Solvent B, 19.01-19.5 min. Column was equilibrated by increasing the flow rate to 0.4 mL/min at 100% B for 19.5-21 min. MS data were acquired using negative and positive ionization in full scan mode over the range of m/z 50-1200. Data were analyzed using Bruker TASQ software version 2.1.22.3. All reported metabolite intensities were normalized to freeze-dried tissue weight, as well as to internal standards with comparable retention times and response in the MS. Metabolite identification was based on a combination of accurate mass, (relative) retention times, ion mobility data and fragmentation spectra, compared to the analysis of a library of standards.

### Lipidomics

The bottom layer, containing the non-polar phase, was transferred to a clean 1.5 mL tube and evaporated under a stream of nitrogen at 60 °C. The residue was dissolved in 100 μL of 1:1 (vol/vol) methanol:chloroform. Lipids were analyzed using a Thermo Scientific Ultimate 3000 binary HPLC coupled to a Q Exactive Plus Orbitrap mass spectrometer. For normal phase separation, 2 μL of each sample was injected onto a Phenomenex® LUNA silica, 250 * 2 mm, 5 µm 100 Å. Column temperature was held at 25 °C. Mobile phase consisted of (A) 85:15 (vol/vol) methanol:water containing 0.0125% (w/volol) formic acid and 3.35 mM ammonia and (B) 97:3 (vol/vol) chloroform:methanol containing 0.0125% formic acid. Using a flow rate of 0.3 mL/min, the LC gradient consisted of: Dwell at 10% A 0-1 min, ramp to 20% A at 4 min, ramp to 85% A at 12 min, ramp to 100% A at 12.1 min, dwell at 100% A 12.1-14 min, ramp to 10% A at 14.1 min, dwell at 10% A for 14.1-15 min. For reversed phase separation, 5 μL of each sample was injected onto a Waters HSS T3 column (150 x 2.1 mm, 1.8 μm particle size). Column temperature was held at 60 °C. Mobile phase consisted of (A) 4:6 (vol/vol) methanol:water and B 1:9 (vol/vol) methanol:isopropanol, both containing 0.1% formic acid and 10 mM ammonia. Using a flow rate of 0.4 mL/min, the LC gradient consisted of: Dwell at 100% A at 0 min, ramp to 80% A at 1 min, ramp to 0% A at 16 min, dwell at 0% A for 16-20 min, ramp to 100% A at 20.1 min, dwell at 100% A for 20.1-21 min. MS data were acquired using negative and positive ionization using continuous scanning over the range of m/z 150 to m/z 2000. Data were analyzed using an in-house developed lipidomics pipeline written in the R programming language (http://ww.r-project.org). All reported lipids were normalized to corresponding internal standards according to lipid class, as well as to freeze-dried tissue weight. Lipid identification was based on a combination of accurate mass, (relative) retention times, fragmentation spectra, analysis of samples with known metabolic defects, and the injection of relevant standards.

# References

1. Warmoes M, Jaspers JE, Pham TV, Piersma SR, Oudgenoeg G, Massink MP, et al. Proteomics of mouse BRCA1-deficient mammary tumors identifies DNA repair proteins with potential diagnostic and prognostic value in human breast cancer. Mol Cell Proteomics. 2012;11(7):M111 013334.

2. Piersma SR, Broxterman HJ, Kapci M, de Haas RR, Hoekman K, Verheul HM, et al. Proteomics of the TRAP-induced platelet releasate. J Proteomics. 2009;72(1):91-109.

3. Pham TV, Piersma SR, Warmoes M, Jimenez CR. On the beta-binomial model for analysis of spectral count data in label-free tandem mass spectrometry-based proteomics. Bioinformatics. 2010;26(3):363-9.

4. Szklarczyk D, Gable AL, Nastou KC, Lyon D, Kirsch R, Pyysalo S, et al. The STRING database in 2021: customizable protein-protein networks, and functional characterization of user-uploaded gene/measurement sets. Nucleic Acids Res. 2021;49(D1):D605-D12.

5. Shannon P, Markiel A, Ozier O, Baliga NS, Wang JT, Ramage D, et al. Cytoscape: a software environment for integrated models of biomolecular interaction networks. Genome Res. 2003;13(11):2498-504.

6. Maere S, Heymans K, Kuiper M. BiNGO: a Cytoscape plugin to assess overrepresentation of gene ontology categories in biological networks. Bioinformatics. 2005;21(16):3448-9.

7. Nepusz T, Yu H, Paccanaro A. Detecting overlapping protein complexes in protein-protein interaction networks. Nat Methods. 2012;9(5):471-2.

8. Heberle H, Meirelles GV, da Silva FR, Telles GP, Minghim R. InteractiVenn: a web-based tool for the analysis of sets through Venn diagrams. BMC Bioinformatics. 2015;16:169.

9. Molenaars M, Schomakers BV, Elfrink HL, Gao AW, Vervaart MAT, Pras-Raves ML, et al. Metabolomics and lipidomics in Caenorhabditis elegans using a single-sample preparation. Dis Model Mech. 2021;14(4).

10. Schomakers BV, Hermans J, Jaspers YRJ, Salomons G, Vaz FM, van Weeghel M, et al. Polar metabolomics in human muscle biopsies using a liquid-liquid extraction and full-scan LC-MS. STAR Protoc. 2022;3(2):101302.

# Supplementary Table 1

| Metabolite | Fold change | Diet effect Q-value |
| --- | --- | --- |
| LysoPC(18:1) | 2.80 | 4.91E-13 |
| LysoPC(20:3) | 2.42 | 7.47E-12 |
| Ergothioneine | 0.37 | 1.09E-09 |
| DL-2-Aminooctanoic acid | 0.24 | 3.57E-09 |
| (D-)4-Hydroxy-2-oxoglutaric acid | 2.27 | 3.02E-08 |
| LysoPE(20:0) LysoPC(17:0) | 1.99 | 3.02E-08 |
| LysoPE(20:1) | 2.31 | 3.21E-08 |
| 3-Indolepropionic acid | 0.41 | 1.83E-07 |
| LysoPC(16:1) | 2.00 | 9.84E-07 |
| Taurocholic acid Tauroursocholic acid Taurallocholic acid Tauro-b-muricholic acid Taurohyocholate | 9.09 | 1.05E-05 |
| m-Aminobenzoic acid 2-Aminobenzoic acid 3-Pyridylacetic acid | 0.31 | 3.05E-05 |
| LysoPC(14:0) | 1.72 | 4.15E-05 |
| 4-(2-Aminophenyl)-2.4-dioxobutanoic acid | 0.42 | 5.63E-05 |
| AC(22:6) | 1.65 | 1.29E-04 |
| Indoleacetaldehyde | 0.66 | 1.68E-04 |
| Uric acid | 1.67 | 2.89E-04 |
| MG(18:2) | 0.53 | 3.42E-04 |
| D-Asparagine Ureidopropionic acid L-Asparagine Glycyl-glycine N-Carbamoylsarcosine | 2.08 | 4.23E-04 |
| Pyrocatechol sulfate | 0.36 | 8.97E-04 |
| Glycerol 3-phosphate Beta-Glycerophosphoric acid | 1.50 | 1.33E-03 |
| Serotonin | 0.68 | 2.01E-03 |
| LysoPC(15:0) LysoPE(18:0) | 1.46 | 2.32E-03 |
| Lanthionine ketimine | 0.36 | 2.33E-03 |
| 2-Hydroxybutyric acid (R)-3-Hydroxybutyric acid (S)-3-Hydroxyisobutyric acid (R)-3-Hydroxyisobutyric acid 3-Hydroxybutyric acid (S)-3-Hydroxybutyric acid 4-Hydroxybutyric acid 2-Methyl-3-hydroxypropanoate | 2.06 | 3.77E-03 |
| L/D-Arginine | 0.42 | 3.92E-03 |
| D-Arginine | 0.42 | 3.92E-03 |
| L-Acetylcarnitine | 0.63 | 4.47E-03 |
| LysoPC(22:6) | 1.40 | 5.30E-03 |
| AC(18:1) | 1.39 | 5.92E-03 |
| LysoPC(18:2) | 1.36 | 7.70E-03 |
| LysoPE(20:4) | 1.38 | 7.70E-03 |
| DG(32:1) | 0.58 | 7.70E-03 |
| LysoPC(20:4) | 1.46 | 1.09E-02 |
| LysoPC(20:4) | 1.46 | 1.10E-02 |
| Phloretin xylosyl-galactoside | 0.78 | 1.14E-02 |
| p-Cresol sulfate | 1.68 | 1.71E-02 |
| Taurochenodesoxycholic acid Tauroursodeoxycholic acid Taurodeoxycholic acid | 2.22 | 1.71E-02 |
| 13.14-Dihydro PGF-1a | 1.64 | 1.71E-02 |
| FA(16:0) TrimethylFA(13:0) | 1.41 | 1.71E-02 |
| CPA(16:0/0:0) | 0.71 | 1.77E-02 |
| 4-Guanidinobutanoic acid | 0.67 | 1.77E-02 |
| N/2-Methylnicotinamide | 0.41 | 1.77E-02 |
| (L-)Glyceric acid | 1.38 | 2.19E-02 |
| 2-Methylacetoacetic acid | 0.72 | 2.37E-02 |
| Hippuric acid | 0.32 | 2.39E-02 |
| AC(22:4) 23-Acetoxysoladulcidine | 1.38 | 3.28E-02 |
| Guanidoacetic acid | 0.70 | 3.28E-02 |
| 5-Hydroxytryptophol 1.2-Dehydrosalsolinol | 0.61 | 3.39E-02 |
| AC(12:1) | 1.50 | 3.66E-02 |
| 3-Chlorotyrosine | 0.55 | 3.84E-02 |
| L-Carnitine | 0.63 | 3.89E-02 |
| 3-Oxo-4.6-choladienoic acid | 0.67 | 3.99E-02 |
| Hypoxanthine | 0.18 | 4.23E-02 |
| 5-Phosphoribosylamine | 0.69 | 4.77E-02 |
| 6.8-Dihydroxypurine Xanthine | 0.25 | 5.38E-02 |
| DihydroxyFA(18:2) EpoxyFA(18:1/0) | 0.74 | 5.40E-02 |
| 3-Hydroxy-2-methyl-[R-(R.S)]-butanoic acid 2-Methyl-3-hydroxybutyric acid 2-Ethylhydracrylic acid 2-Hydroxy-3-methylbutyric acid 3-Hydroxy-2-methyl-[S-(R.R)]-butanoic acid 3-Hydroxyvaleric acidErythronilic acid 3-Hydroxyisovaleric acid 2-Hydroxyvaleric acid 2-Hydroxy-2-methylbutyric acid 4-Hydroxyisovaleric acid | 1.40 | 5.90E-02 |
| Threonic acid | 1.29 | 6.52E-02 |
| Glycyl-Lysine Lysyl-Glycine | 1.43 | 7.48E-02 |
| (S)-3.4-Dihydroxybutyric acid 2.4-Dihydroxybutanoic acid 4-Deoxyerythronic acid 4-Deoxythreonic acid Erythrose L-Erythrulose | 1.39 | 7.84E-02 |
| AC(18:0) | 1.43 | 7.96E-02 |
| Indole-3-carboxilic acid-O-sulphate | 0.67 | 7.97E-02 |
| Sulfolithocholylglycine | 1.91 | 8.13E-02 |
| N-Acetylornithine  Glycyl-Valine  Valyl-Glycine | 0.77 | 8.13E-02 |
| Galactonic acid  Gluconic acid  Gulonic acid | 1.77 | 8.13E-02 |
| FA(16:1) | 1.34 | 8.26E-02 |
| LysoPC(20:5) | 1.32 | 8.68E-02 |
| FA(18:2) | 0.71 | 8.68E-02 |
| 3-Hydroxyquinine | 0.78 | 8.99E-02 |
| MG(20:4) | 0.68 | 9.33E-02 |
| D-Glucuronic acid  Galacturonic acid  Iduronic acid  3-Dehydro-L-gulonate  5-Keto-D-gluconate  2-Keto-L-gluconate | 1.56 | 9.71E-02 |
| 3b-Hydroxy-5-cholenoic acid | 0.80 | 9.71E-02 |

**Supplementary Table 1. Effect of Western diet-feeding on metabolites in serum of normal chow and Western diet-fed mice.** Fold change indicates abundance in WD versus NC-fed animals, regardless of genotype. Data were analyzed using R-limma and p-values were corrected for multiple testing via the Benjamini-Hochberg FDR procedure. PC indicates phosphatidylcholine; PE, phosphatidylethanolamine; AC, acylcarnitine; MG, monoglyceride; DG, diglyceride; FA, fatty acid; CPA, cyclic phosphatidic acid. N=8 individual mouse serum samples per group.

# Supplementary Table 2

| Total proteins identified (n) | | 3797 |  |  |
| --- | --- | --- | --- | --- |
| Total proteins included (n) | | 2552 |  |  |
| Group comparison | | Number of significantly different proteins (p<0.05) | | |
|  |  | Total | Higher abundance | Lower abundance |
| WT NC | HET NC | 22 | 8 | 14 |
| WT NC | WT WD | 56 | 43 | 13 |
| HET NC | HET WD | 64 | 48 | 16 |
| WT WD | HET WD | 41 | 8 | 33 |

**Supplementary Table 2. Descriptive summary of proteins identified via mass spectrometry-based proteomics and group comparisons that were made.** WT indicates wild-type; HET, heterozygous; NC, normal chow; WD, Western diet. WT Normal chow N=7; HET Normal chow N=8; WT Western diet N=8; HET Western diet N=8 individual left ventricular cardiac tissue samples.

# Supplementary Figure 1


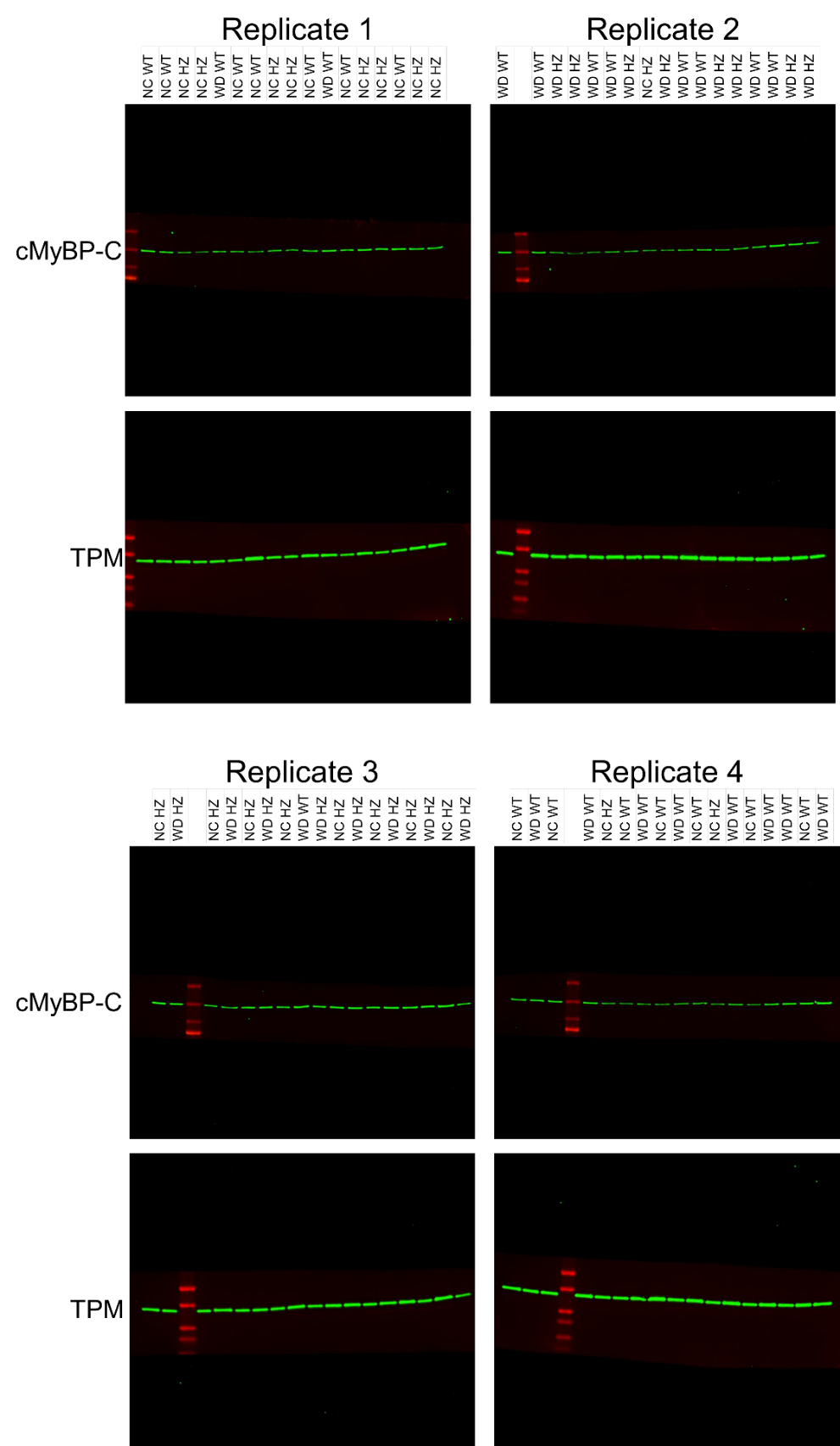


**Supplementary Figure 1. Full-size blots of cardiac myosin-binding protein C (cMyBP-C) and tropomyosin (TPM).** Images were captured using fluorescence detection, and relate to panels 2F,G. NC indicates normal chow; WD, Western diet; WT, wild-type; HET, heterozygous

# Supplementary Figure 2


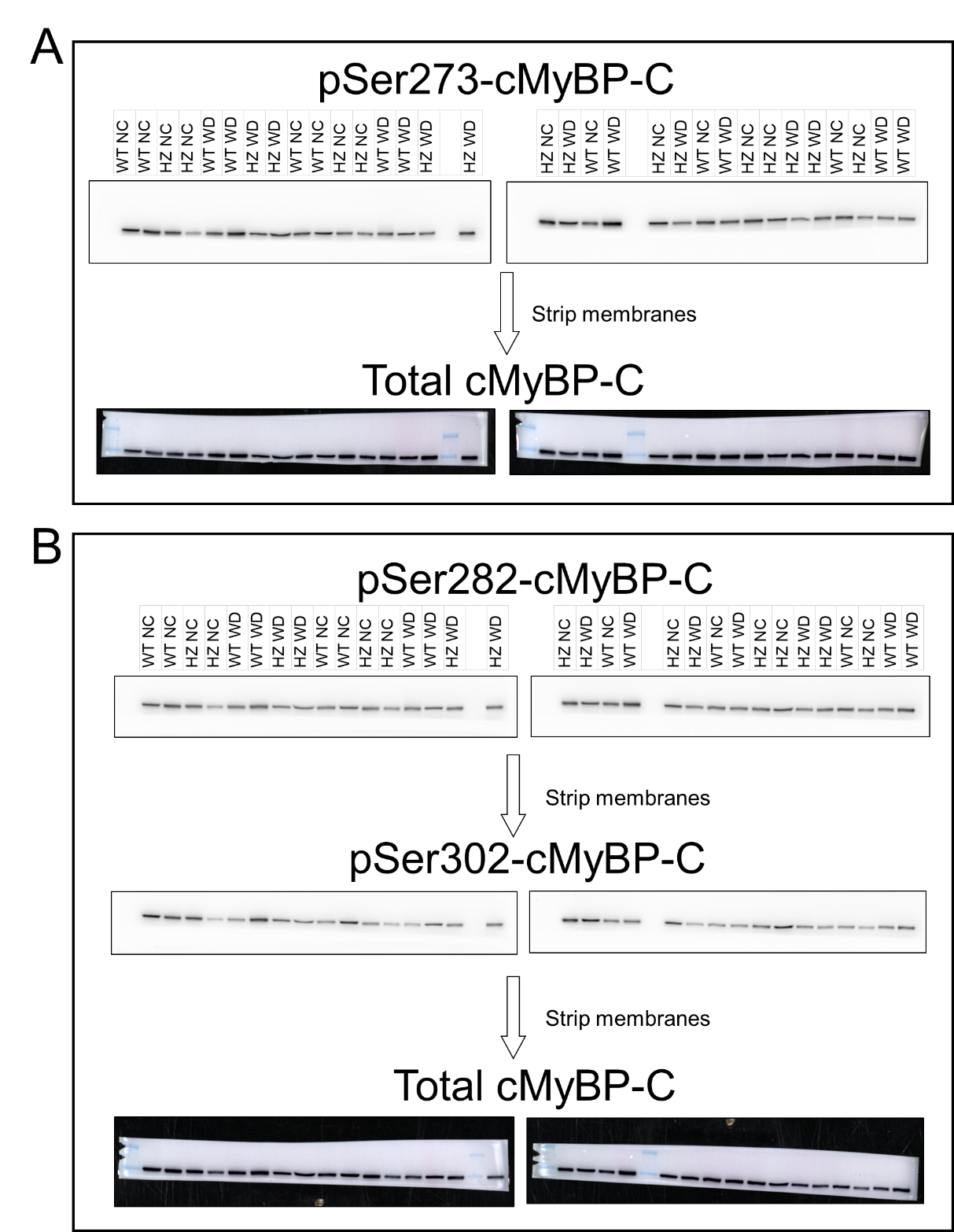


**Supplementary Figure 2. Full-size blots of phosphorylated and total cardiac myosin-binding protein C (cMyBP-C). I**mages were captured using electrochemiluminescence, and relate to panels 2H,I. **(A)** shows phospho-Ser273 cMyBP-C and total cMyBP-C as reference; **(B)** shows phospho-Ser282 and phospho-Ser302 and total cMyBP-C as reference. NC indicates normal chow; WD, Western diet; WT, wild-type; HET, heterozygous

# Supplementary Figure 3


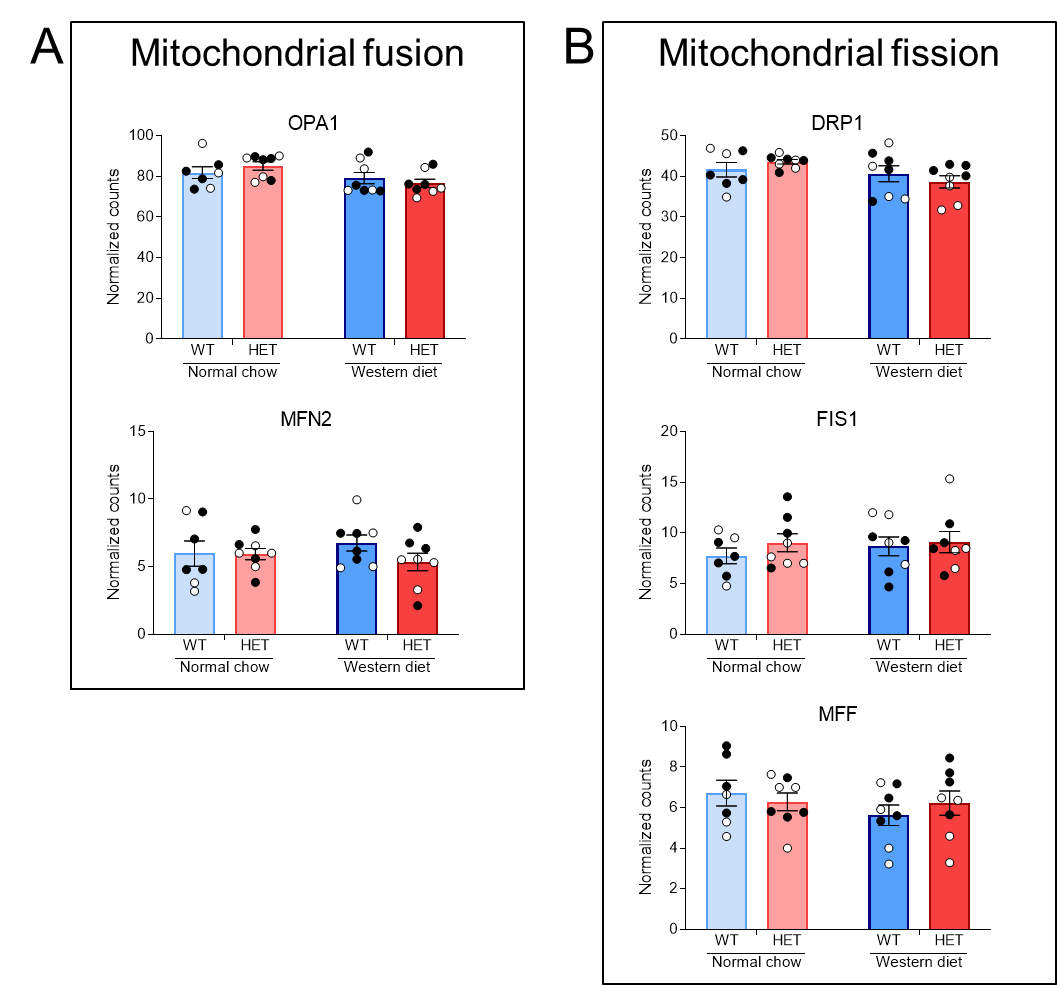


**Supplementary Figure 3. Levels of proteins involved in mitochondrial fusion and mitochondrial fission are unaltered between experimental groups.** Panels A and B show proteomics data of left ventricular cardiac samples from wild-type (WT) and heterozygous (HET) mice that received normal chow or a Western diet. **(A)** shows data of mitochondrial fusion-related proteins OPA1 and MFN2; **(B)** shows data of mitochondrial fission-related proteins DRP1, FIS1 and MFF. Data are expressed as mean ± standard error of the mean. Group differences in protein abundance were analyzed via beta-binominal statistics. WT Normal chow N=7; HET Normal chow N=8; WT Western diet N=8; HET Western diet N=8 individual left ventricular cardiac tissue samples. White and black symbols indicate data from male and female mice, respectively.

# Supplementary Figure 4


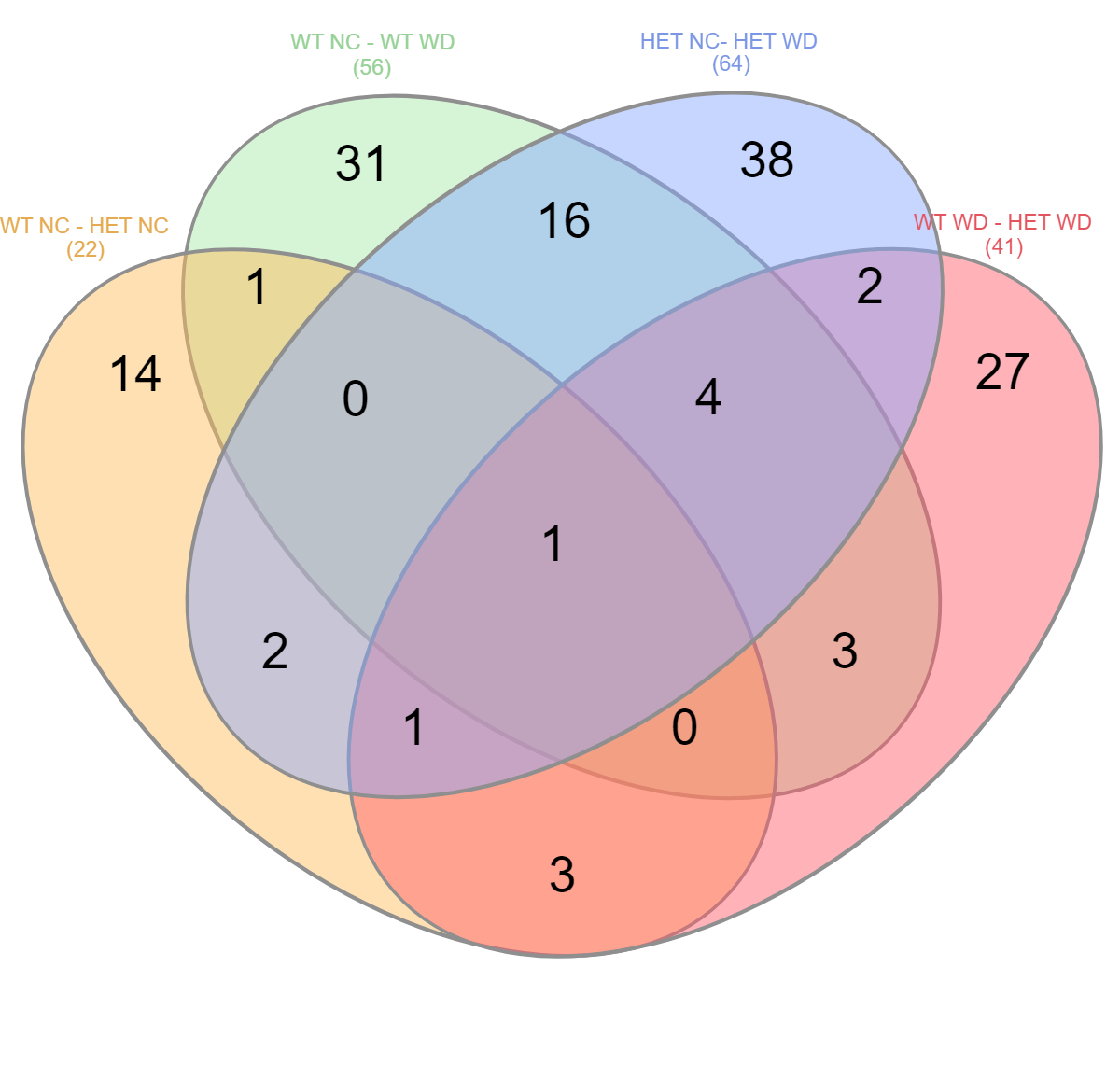


**Supplementary Figure 4. Venn diagram showing the amount of overlap in differentially abundant proteins between group comparisons.** Group differences in protein abundance were analyzed via beta-binominal statistics. WT indicates wild-type; HET, heterozygous; NC, normal chow; WD, Western diet. WT Normal chow N=7; HET Normal chow N=8; WT Western diet N=8; HET Western diet N=8 individual left ventricular cardiac tissue samples.

# Supplementary Table 3

| WT WD vs WT NC | | |  | HET WD vs HET NC | | |  | HET WD vs WT WD | | |
| --- | --- | --- | --- | --- | --- | --- | --- | --- | --- | --- |
| Protein | FC | P-value |  | Protein | FC | P-value |  | Protein | FC | P-value |
| Cluster #1 | | |  | Cluster #1 | | |  | ACADM | -1.14 | 6.0E-04 |
| ABCD3 | 1.52 | 5.2E-03 |  | ABCD1 | 2.84 | 2.0E-02 |  | ACADVL | -1.13 | 5.5E-04 |
| ACAD11 | 1.30 | 2.1E-02 |  | ABCD3 | 1.59 | 1.8E-03 |  | ACOT2 | -1.22 | 1.4E-03 |
| ACADL | 1.08 | 3.3E-02 |  | ACAD11 | 1.29 | 3.4E-02 |  | ACOX1 | -1.22 | 1.8E-02 |
| ACADVL | 1.18 | 3.7E-05 |  | ACADVL | 1.11 | 1.9E-03 |  | CPT1B | -1.10 | 3.4E-02 |
| ACOT1 | 2.28 | 1.3E-02 |  | ACOT1 | 2.23 | 1.9E-02 |  | CPT2 | -1.13 | 5.9E-03 |
| ACOT2 | 1.39 | 4.1E-05 |  | ACOT2 | 1.16 | 2.1E-02 |  | CRAT | -1.14 | 1.3E-02 |
| ACOX1 | 1.67 | 1.8E-05 |  | ACOX1 | 1.67 | 4.9E-04 |  | EHHADH | -1.51 | 2.6E-02 |
| ACSF2 | 1.50 | 7.7E-03 |  | APOA4 | 1.61 | 1.3E-05 |  | PLIN5 | -1.66 | 4.6E-02 |
| ALDH2 | -1.18 | 1.3E-02 |  | CAT | 1.37 | 2.8E-04 |  |  |  |  |
| BDH1 | -1.36 | 7.0E-03 |  | DBI | 1.44 | 7.5E-04 |  |  |  |  |
| CAT | 1.28 | 1.2E-02 |  | EHHADH | 1.93 | 1.1E-02 |  |  |  |  |
| CPT2 | 1.14 | 4.7E-03 |  | EPHX2 | 1.26 | 3.9E-03 |  |  |  |  |
| DBI | 1.32 | 3.4E-02 |  | FABP3 | 1.17 | 9.9E-03 |  |  |  |  |
| ECH1 | 1.22 | 1.7E-02 |  | FABP4 | 1.16 | 4.5E-03 |  |  |  |  |
| EHHADH | 2.00 | 1.1E-03 |  | HSD17B4 | 1.31 | 9.6E-03 |  |  |  |  |
| EPHX2 | 1.21 | 1.4E-02 |  | HSDL2 | 1.17 | 2.7E-02 |  |  |  |  |
| FABP5 | 1.25 | 4.9E-02 |  | IVD | -1.23 | 7.7E-03 |  |  |  |  |
| PDK4 | 2.23 | 5.4E-05 |  | PCCA | -1.13 | 1.8E-02 |  |  |  |  |
| SCP2 | 1.24 | 2.4E-02 |  | PDK4 | 1.61 | 7.0E-04 |  |  |  |  |
| UCP3 | 1.59 | 1.3E-02 |  | PDPR | -1.30 | 4.9E-02 |  |  |  |  |
|  |  |  |  | PHYH | 1.67 | 3.2E-02 |  |  |  |  |
| Cluster #2 | | |  | PLIN2 | -1.59 | 4.8E-02 |  |  |  |  |
| FBP2 | 2.27 | 1.2E-02 |  | RBP1 | 1.92 | 3.8E-02 |  |  |  |  |
| MDH1 | -1.10 | 1.9E-02 |  | SCP2 | 1.36 | 1.5E-03 |  |  |  |  |
| ME1 | 1.66 | 2.0E-03 |  | THNSL1 | -1.64 | 3.7E-02 |  |  |  |  |
| PCX | -1.38 | 1.0E-03 |  | UCP3 | 2.05 | 6.1E-04 |  |  |  |  |
| PDHB | -1.17 | 1.0E-03 |  |  |  |  |  |  |  |  |
|  |  |  |  | Cluster #2 | | |  |  |  |  |
|  |  |  |  | ALDOB | -2.19 | 1.3E-03 |  |  |  |  |
|  |  |  |  | FBP2 | 2.25 | 1.7E-02 |  |  |  |  |
|  |  |  |  | PYGB | -1.15 | 1.2E-03 |  |  |  |  |
|  |  |  |  | PYGM | -1.12 | 1.4E-03 |  |  |  |  |
|  |  |  |  | PCCA | -1.13 | 1.8E-02 |  |  |  |  |
|  |  |  |  | PCX | -1.24 | 2.2E-02 |  |  |  |  |
|  |  |  |  | OGDH | -1.10 | 1.2E-02 |  |  |  |  |

**Supplementary Table 3. Significantly different interacting proteins (according to STRING database) for each group comparison.** Group differences in protein abundance were analyzed via beta-binominal statistics. WT indicates wild-type; HET, heterozygous; NC, normal chow; WD, Western diet; FC, fold change. WT Normal chow N=7; HET Normal chow N=8; WT Western diet N=8; HET Western diet N=8 individual left ventricular cardiac tissue samples.
